# Advanced Cancer Immunotherapy via SMARCAL1 Blockade Using a Glucose‐Responsive CRISPR Nanovaccine

**DOI:** 10.1002/advs.202502929

**Published:** 2025-07-12

**Authors:** Yuwei Li, Yuanyi Zhang, Chenchen Li, Guoping Chen, Pir Muhammad, Yonghong Yao, Lifang Gao, Zhigang Liu, Yanli Wang

**Affiliations:** ^1^ Engineering Research Center of Tropical Medicine Innovation and Transformation of Ministry of Education International Joint Research Center of Human‐machine Intelligent Collaborative for Tumor Precision Diagnosis and Treatment of Hainan Province Hainan Academy of Medical Sciences Hainan Medical University Hainan 571199 China; ^2^ Department of Immunology & Key Laboratory of Tropical Translational Medicine of Ministry of Education School of Basic Medicine and Life Sciences Hainan Medical University Hainan 571199 China; ^3^ State Key Laboratory of Respiratory Disease for Allergy School of Medicine Shenzhen University Shenzhen 518060 China

**Keywords:** glucose‐responsive, immunotherapy, nanovaccine, opposing functions of interferon, SMARCAL1, STING

## Abstract

Cancer immunotherapy that activates the stimulator of interferon genes (STING) signaling pathway to resist tumors has recently attracted considerable attention. However, STING activation can induce opposing interferon functions that contribute to T‐cell exhaustion via programmed death‐ligand 1 (PD‐L1). In particular, effectively using the immune system to combat tumors remains a substantial challenge due to tumor immunosuppressive factors such as SMARCAL1. Here, a glucose‐responsive CRISPR nanovaccine is developed for enhancing STING signaling while inhibiting interferon‐mediated immunosuppressive feedback. The formulation encapsulates a bimetallic zeolitic imidazolate framework with glucose oxidase (GOx) and CRISPR‐mediated SMARCAL1 gene‐editing plasmids. The dual enzyme‐driven cascade reactions of peroxidase and GOx generate reactive oxygen species (ROS) and gluconic acid, which release and activate the genome‐editing system. The silencing of *SMARCAL1* enhances STING activity and inhibits PD‐L1 expression, resulting in the termination of PD‐L1‐mediated opposing functions of interferon. Zinc ions and double‐stranded DNA formed via ROS further activate the STING pathway, effectively inducing dendritic cell maturation and immune system activation. This is a critical report of in situ CRISPR nanovaccination driven by dual enzymes. The work highlights the potential of glucose‐responsive CRISPR nanovaccination in bolstering antitumor immunity and extends the implementation of gene editing in cancer immunotherapy.

## Graphical abstract

A glucose‐catalyzed chemodynamic strategy is proposed to activate therapeutic genome editing of SMARCAL1to improve cancer immunotherapy. This innovative design featuring Fenton reaction activity and glucose consumption ability, not only effectively synergizes to activate the cGAS‐STING pathway but also inhibits the opposing functions of interferon by suppressing PD‐L1 expression, providing a potent antitumorigenic gene‐editing therapy.

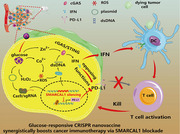

## 1 Introduction

Immunotherapies, including cancer vaccines, immune cell activation, and immune checkpoint blockade, have become a mainstay in the treatment of various cancers.^[^ ^1^ ^]^ The immune system comprises innate and adaptive systems,^[^ ^2^ ^]^ and the capacity of these systems to interact with each other is crucial for enhancing the human immune system's cytotoxic effects in malignancy treatment.^[^ ^3^ ^]^ Tumor‐derived cyclic GMP‐AMP synthase (cGAS) triggers the stimulator of interferon genes (STING) signaling pathway to promote the development and function of innate and adaptive immunity.^[^ ^4^ ^]^ Upon activation, the STING signaling pathway can induce the production of type I interferons, which are responsible for bridging innate and adaptive immunity^[^ ^5^ ^]^ and initiating immune responses by enhancing the phagocytic and antigen‐presenting capabilities of dendritic cells (DCs) and macrophages against tumors.^[^ ^6^ ^]^ However, recent studies have shown that activation of the STING pathway can lead to an opposing effect from type I interferons, which may drive T‐cell exhaustion through programmed death‐ligand 1 (PD‐L1).^[^ ^7^ ^]^

Tumor cells usually employ a multitude of immunosuppressive strategies to evade immune destruction. SWI/SNF‐related, matrix‐associated, actin‐dependent regulator of chromatin, subfamily A‐like 1 (SMARCAL1), a component of the ATP‐dependent chromatin remodeling protein SNF2 family,^[^ ^8^ ^]^ plays a critical role in tumor migration, invasion, metastasis, and immune escape and is overexpressed in various tumor types.^[^ ^9^ ^]^ SMARCAL1 facilitates the immune protection of tumors through a dual mechanism.^[^ ^10^ ^]^ By maintaining genome stability and limiting endogenous DNA damage, SMARCAL1 inhibits the cGAS‐STING‐dependent immune signaling pathway during cancer cell growth, helping cancer cells evade recognition and clearance by the immune system.^[^ ^11^ ^]^ SMARCAL1 also collaborates with Jun proto‐oncogene protein (JUN), a member of the AP‐1 family, to maintain chromatin accessibility at the PD‐L1 transcriptional regulatory site, thereby promoting PD‐L1 expression in cancer cells and assisting in immune evasion through PD‐L1‐mediated immune checkpoint responses.^[^ ^10^ ^]^ SMARCAL1 has emerged as a critical mediator in the immune evasion strategies employed by tumors, and silencing SMARCAL1 represents a novel approach for efficiently improving antitumor immune responses.

**Figure 1:**
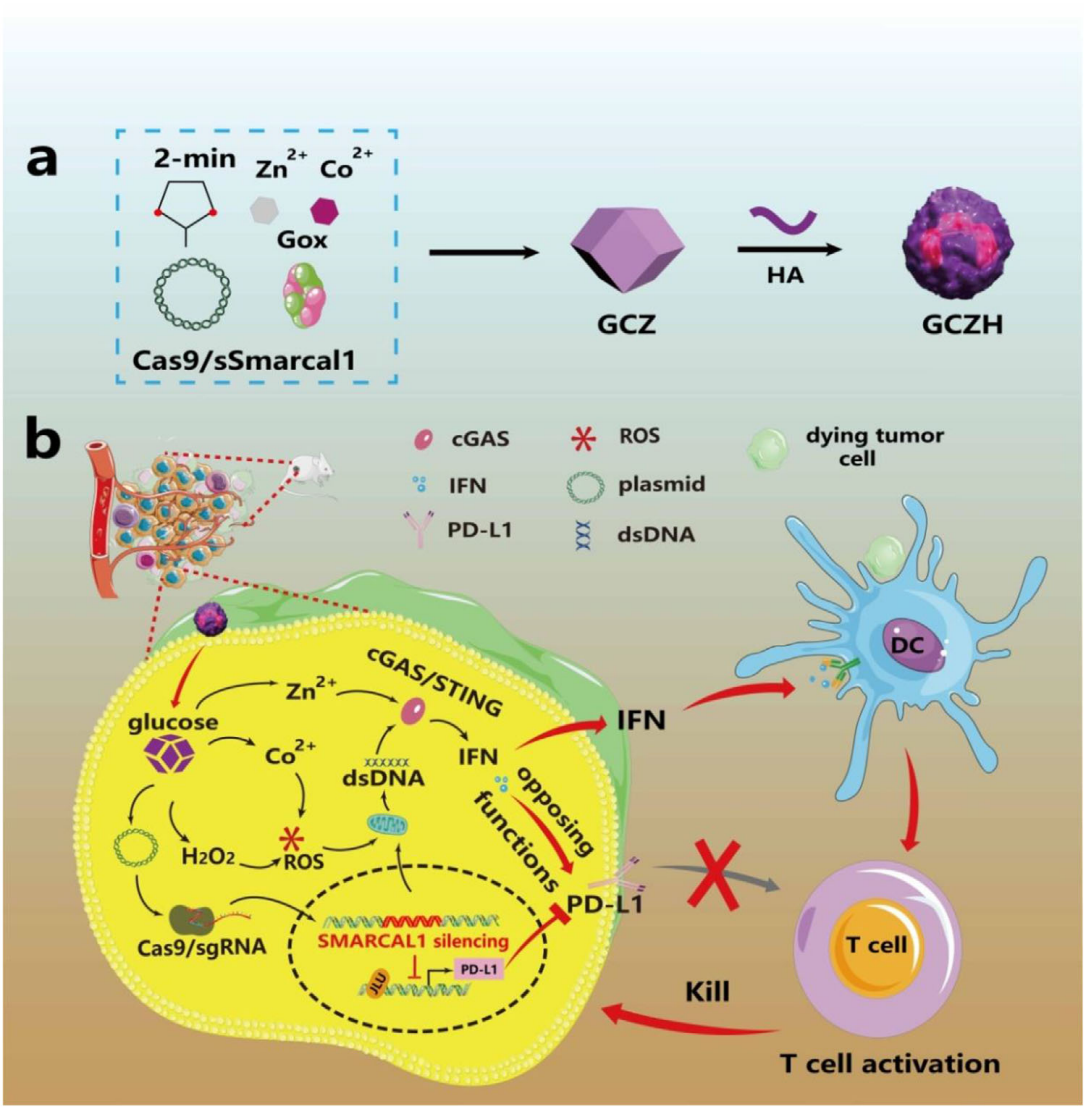
Schematic illustration of glucose‐responsive gene editing of SMARCAL1 for synergistically enhancing STING‐mediated cancer immunotherapy. a) Preparation of the glucose‐responsive gene editor. b) Synergistic strategy to enhance cGAS‐STING signaling and PD‐L1 inhibition through glucose‐responsive genome editing combined with SMARCAL1 blockade.

The CRISPR/Cas9 system, which uses clustered regularly interspaced short palindromic repeats (CRISPR)‐associated protein 9 (Cas9), has emerged as a promising gene‐editing method for treating various diseases through cellular reprogramming.^[^ ^12^ ^]^ Efficient intracellular delivery of the CRISPR/Cas9 machinery is a prerequisite for successfully disrupting tumor evolution genes.^[^ ^13^ ^]^ Zeolitic imidazolate frameworks (ZIFs), a family of metal‒organic frameworks (MOFs), have been reported as effective CRISPR/Cas9 delivery nanocarriers.^[^ ^14^ ^]^ Bioresponsive nanocarriers that control the release of Cas9/sgRNA in a biologically specific manner, such as through pH, redox conditions, or small physiological molecules, could significantly improve targeted genome editing.^[^ ^15^ ^]^ Here, we propose a glucose‐catalyzed chemodynamic method to trigger the gene knockout of SMARCAL1, augmenting STING signaling and inhibiting the opposing functions of type I interferons for cancer immunotherapy. As illustrated in **Figure** **1**, ZnCo‐ZIF‐encapsulated genome editing plasmids and glucose oxidase (GOx) were coated with hyaluronic acid (HA) to target cancer cells. HA can specifically bind to the HA receptor (CD44), which is a cell surface adhesion molecule that is overexpressed on multiple types of cancer cells, including colorectal, ovarian, gastric, pancreatic, head and neck, and breast cancer.^[^ ^16^ ^]^ GOx catalyzes the oxidation of glucose to produce H_2_O_2_, which is then broken down into hydroxyl radicals (•OH) by Co, leading to an increase in double‐stranded DNA (dsDNA) levels. The SMARCAL1 gene is knocked out by the CRISPR/Cas9 genome editing plasmid, resulting in further elevation of dsDNA, which improves the activation of the cGAS‐STING pathway with the assistance of Zn. Moreover, blocking SMARCAL1 inhibits PD‐L1 expression and enhances the recognition and destruction of tumors by tumor‐specific T cells. The synergy between the inhibition of PD‐L1 expression and the activation of cGAS‐STING could induce potent antitumor immune responses and ultimately suppress tumor growth. This work paves the way for activating tumor immunotherapy through the combination of gene editing and chemodynamic therapy.

## 2 Results and Discussion

### 2.1 Construction and Characterization of the Nanoparticles

**Figure 2:**
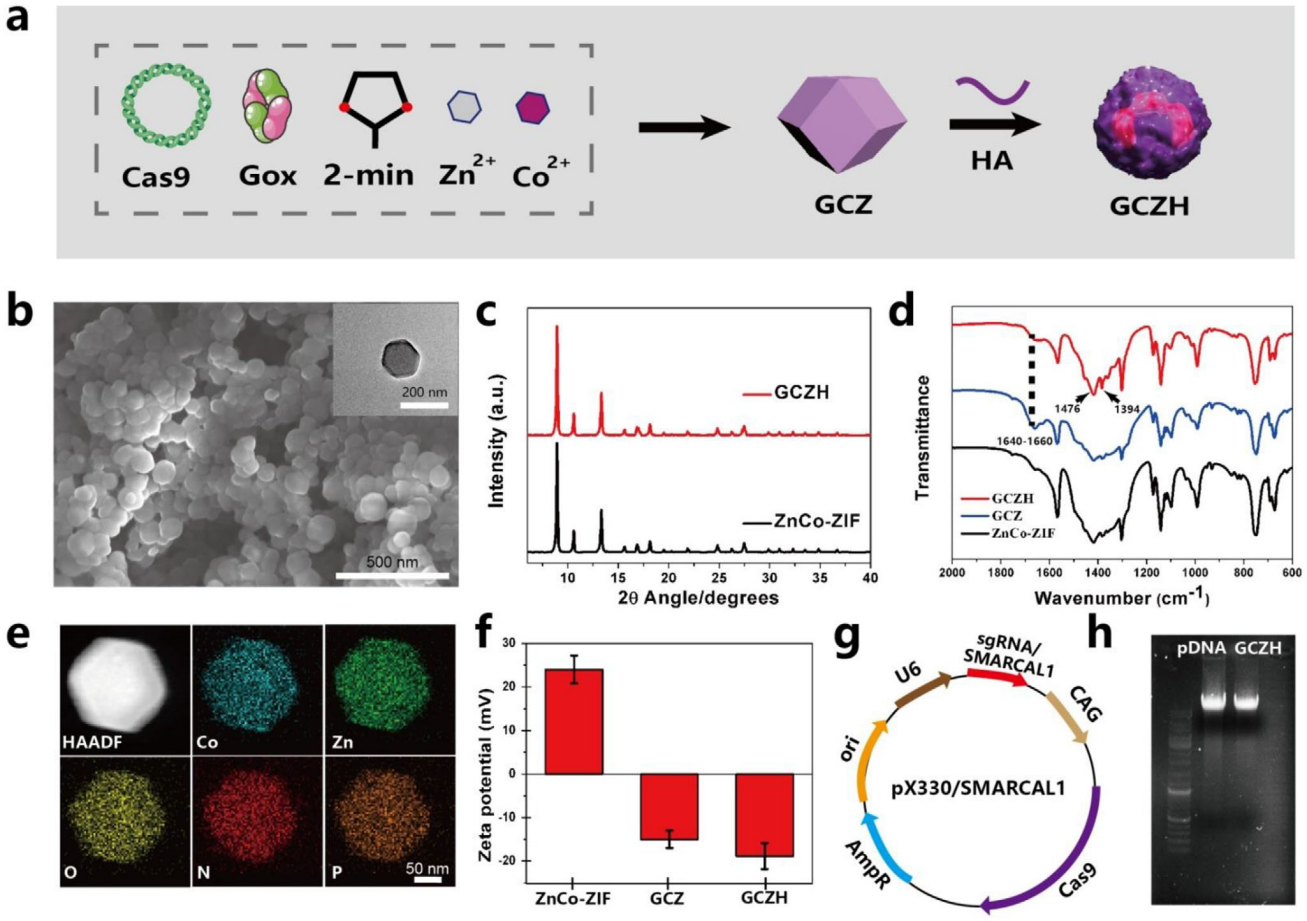
Characterization of the GCZH NPs. a) Schematic illustration of the preparation of the GCZH NPs. b) SEM and TEM (inset) images of the GCZH NPs. c) XRD pattern of the ZnCo‐ZIF and GCZH NPs. d) FT‐IR spectra of the ZnCo‐ZIF, GCZ, and GCZH NPs. e) Corresponding area‐element mappings of the GCZH NPs. f) Zeta potentials of ZnCo‐ZIF, GCZ, and GCZH (mean ± SD, *n* = 3). g) Schematic structure of the pX330 vector after the insertion of the sgRNA targeting SMARCAL1 into the CRISPR/Cas9 plasmid. h) Gel electrophoresis assay of naked pDNA and GCZH.

Nanoparticles (NPs) were synthesized via a simple one‐step process (**Figure** 1a). Cobalt chloride hexahydrate, zinc nitrate hexahydrate, and plasmids were dissolved in water to form solution A, and 2‐methylimidazole was mixed with GOx to yield solution B. To generate GOx‐cas9@ZnCo‐ZIF (hereafter referred to as GCZ), solutions A and B were mixed and strongly agitated at room temperature. ZnCo‐ZIF and the plasmids in ZnCo‐ZIF and GOx in ZnCo‐ZIF were prepared as controls via the same approach. Post‐synthesis, hyaluronic acid (HA) was adsorbed on GOx@ZnCo‐ZIF, Cas9@ZnCo‐ZIF, and GOx‐cas9@ZnCo‐ZIF (hereafter referred to as GCH, GZH, and GCZH, respectively). Scanning electron microscopy (SEM) and transmission electron microscopy (TEM) revealed that the GCZH NPs exhibited a rhombic dodecahedral shape with an average diameter of ≈160 nm (Figure 1b). Dynamic light scattering also revealed that the particle size of GCZH was ≈110 nm, with a narrow size distribution (Figure S1, Supporting Information). The phase purity of the crystallinity was investigated via X‐ray diffraction (XRD), with the pattern for GCZH closely matching that of ZnCo‐ZIF (Figure 1c; Figure S2, Supporting Information), indicating that the incorporation of GOx and plasmids did not affect the crystal structure. The encapsulation of HA was assessed via Fourier transform infrared (FTIR) spectroscopy. As shown in Figure 1d, the characteristic absorption peaks of the FTIR spectrum were observed at 1394 and 1476 cm^−1^, which can be attributed to the absorption of the amide II and III bands of HA.^[^ ^17^ ^]^ Compared with those of the GCZ sample, the presence of these peaks in the GCZH sample confirmed successful HA encapsulation. The peaks in the FTIR spectrum at ≈1640–1660 cm^−1^ were attributed primarily to the C═O stretching mode,^[^ ^18^ ^]^ which indicated the presence of GOx in the GCZH sample. The presence of zinc and cobalt in the GCZH sample was confirmed via inductively coupled plasma (ICP) analysis, which revealed a Zn:Co ratio of 55:45 (Figure S3, Supporting Information), slightly deviating from the intended 50:50 ratio during synthesis. Elemental mapping further demonstrated that cobalt and zinc were uniformly distributed throughout the GCZH framework (Figure 1e), suggesting successful bimetallic MOF formation. Nitrogen adsorption‒desorption isotherms confirmed the encapsulation of GOx and plasmids within GCZH, as evidenced by a reduction in the BET surface area (Figure S4, Supporting Information). The GOx loading efficiency, which was calculated via the BCA method (Figure S5, Supporting Information), was 86% (≈10 wt.%), which was in accordance with the thermogravimetric analysis (TGA) results (Figure S6, Supporting Information). Additionally, the ζ‐potential values of ZnCo‐ZIF (+20.1 mV), GCZ (−13.8 mV), and GCZH (−18.4 mV) revealed the successful encapsulation of HA, GOx, and pDNA into the GCZH framework (Figure 1f). A specific sgRNA targeting SMARCAL1 via CRISPR/Cas9 was designed, successfully subcloned, and inserted into the pX330 plasmid for efficient genome editing at the SMARCAL1 locus (Figure 1g; Figure S7, Supporting Information). Agarose gel electrophoresis confirmed plasmid encapsulation within the GCZH NPs, with a substitution rate of 6.5% (Figure 1h). GCZH samples were collected over 30 days, and the particle size and ζ‐potential were evaluated. As shown in Figure S8 (Supporting Information), there were no significant changes or deviations in size or zeta potential over the 1‐month period. The evaluated results showed that GCZH had good colloidal stability. Collectively, these results confirm the successful synthesis of the GCZH NPs. **Figure** **2**

### 2.2 Biochemical and Spectral Characterization of the GCZH NPs In Vitro

**Figure 3:**
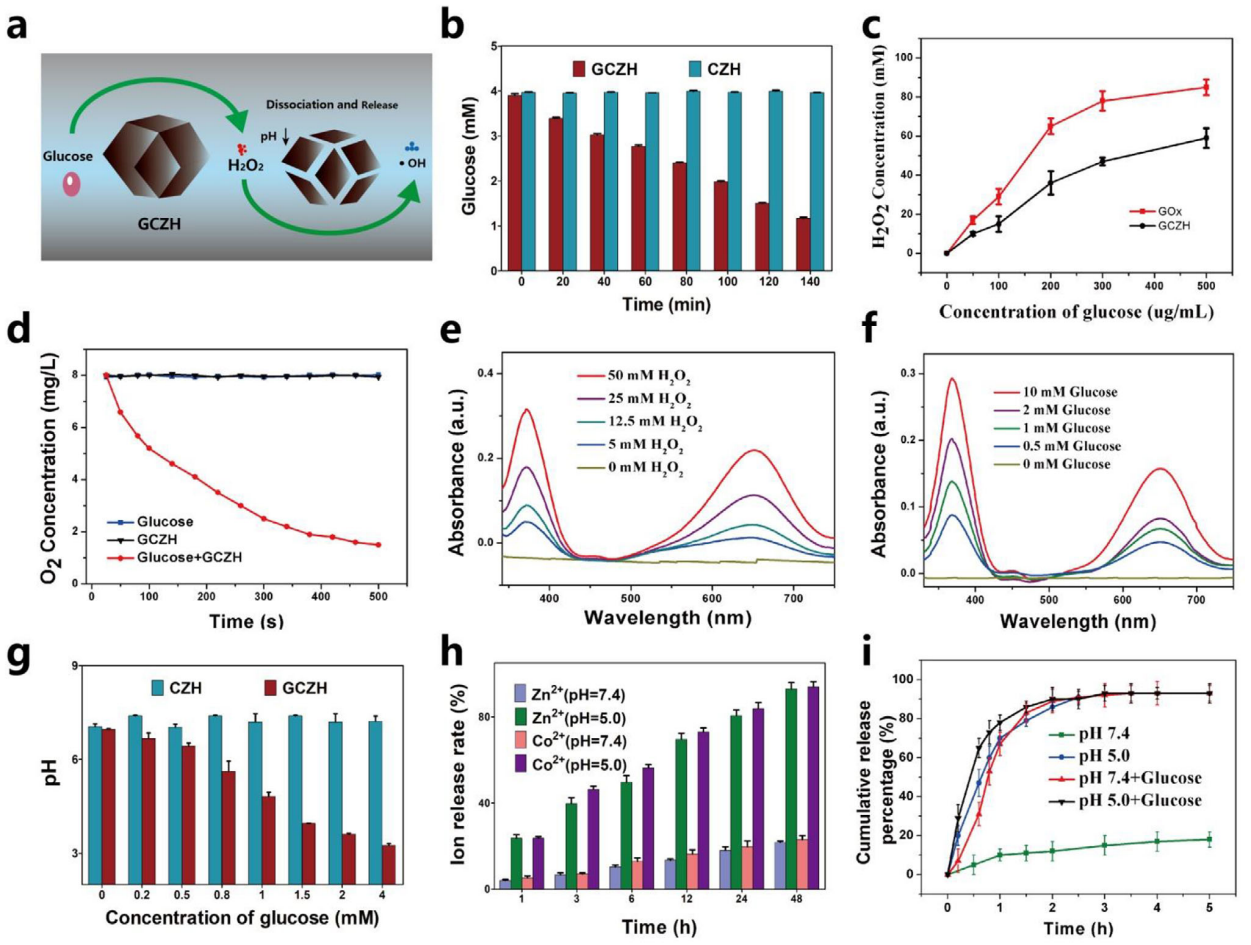
The catalytic activity and pH‐response release of the GCZH NPs. a) Illustration of the cascade catalytic mechanism of the GCZH NPs. b) Consuming glucose ability of the GCZH NPs (mean ± SD, *n* = 3). c) H_2_O_2_ generation ability of the GCZH and GOx (mean ± SD, *n* = 3). d) O_2_ consumption of the GCZH solution upon the addition of glucose. e,f) UV–vis spectrum of GCZH at different concentrations of H_2_O_2_ (e) and glucose (f). g) pH values of the GCZ and GCZH solutions in the absence of glucose (mean ± SD, *n* = 3). h) Ion release at various times and under various conditions (mean ± SD, *n* = 3). i) The release of Cas9/sgSMARCAL1 pDNA from GCZH in different medias (mean ± SD, *n* = 3).

The metabolic pathway of tumor cells predominantly relies on aerobic glycolysis, a phenomenon known as the Warburg effect.^[^ ^19^ ^]^ This effect is characterized by a high intracellular glucose concentration in tumor cells.^[^ ^20^ ^]^ The catalytic action of GOx can effectively deplete glucose while simultaneously increasing acidity, hypoxia, and hydrogen peroxide (H_2_O_2_) levels within tumor cells.^[^ ^21^ ^]^ The resulting intratumoral acidity can trigger the sustained release of drugs from pH‐sensitive carriers, providing a strategic approach for drug delivery.^[^ ^22^ ^]^ Moreover, the depletion of glucose can effectively reduce the energy supply to tumor cells, leading to their starvation and subsequent death, suggesting a potent starvation strategy for cancer therapy.^[^ ^23^ ^]^ In this study, GOx was encapsulated within a well‐designed NP for a cascade reaction to consume glucose (**Figure** **3**a). The catalytic potential of the GCZH composite was subsequently examined. Notably, the glucose‐consuming ability of GCZH was comparable to that of free GOx at equivalent concentrations, as shown in Figure 3b, indicating that our nanoplatform retained the high enzymatic activity characteristic of GOx. The glucose oxidation process catalyzed by GOx generates H_2_O_2_, with the yield increasing as the glucose concentration increases (Figure 3c), which was further quantified via an H_2_O_2_ assay kit. The dissolved oxygen concentrations decreased during the glucose oxidation process (Figure 3d). As depicted in Figure 3e and Figure S9 (Supporting Information), GCZH demonstrated a remarkable ability to catalyze the oxidation of the peroxidase substrate 3,3′,5,5′‐tetramethylbenzidine (TMB), highlighting the peroxidase‐like activity of this nanoplatform. Furthermore, the feasibility of a cascade reaction mediated by GCZH was explored, with the rate of TMB oxidation increasing progressively as the glucose concentration increased, as shown in Figure 3f, elucidating the generation of hydroxyl radicals (•OH) through the cascade reaction between GCZH and glucose. Drawing on the GOx‐mediated conversion of glucose into gluconic acid, we observed a significant drop in pH in the presence of glucose and GCZH, as illustrated in Figure 3g and Figure S10 (Supporting Information). The dissociation of the ZnCo‐ZIF NPs was accelerated under acidic conditions. This could accelerate the release of cobalt, zinc, and plasmid from the GCZH NPs. As depicted in Figure 3h, cobalt and zinc were released quickly from the GCZH NPs at pH 5.0. More than 80% of the plasmid was released within 2 h at pH 5.0 (Figure 3i). However, only 10% of the cobalt, zinc, and plasmid were released at pH 7.4. The increased acidity is responsible for the decomposition of GCZH, thereby facilitating the release of the plasmid. These results suggest that the cascade catalytic GCZH could reduce glucose levels effectively and accomplish glucose‐responsive dual enzyme‐driven plasmid release.

### 2.3 Glucose‐Responsive SMARCAL1 Gene Knockout and Activation of the STING Signaling Pathway

**Figure 4:**
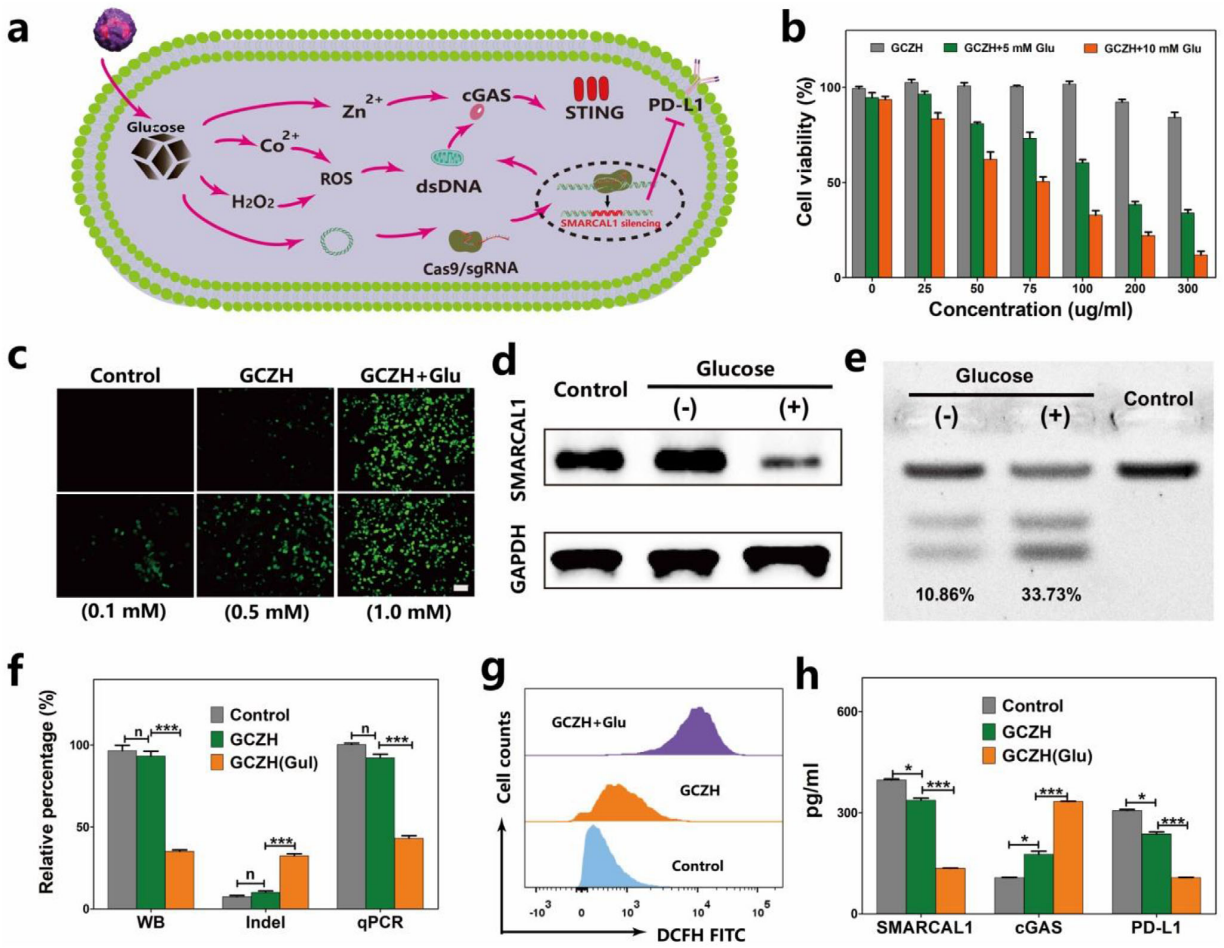
Glucose‐responsive silencing of the SMARCAL1 gene by the GCZH effectively activated the STING signaling pathway. a) Schematic representation of glucose‐enhanced SMARCAL1 knockout and activation of the STING signaling pathway. b) The relative viability of 4T1 cells incubated with different concentrations of GCZH under different concentrations of glucose (mean ± SD, *n* = 3). c) Effect of glucose on the expression of the plasmid. The GFP expression of the transfected cells was evaluated under different treatment conditions. The scale bar is 50 µm. d) Western blot analysis of SMARCAL1 expression in 4T1 cells after different treatments. The level of protein loaded was evaluated with respect to the level of GAPDH. e) T7EI cleavage assays of SMARCAL1 gene disruption after various treatments. f) Relative proportion of SMARCAL1 expression analyzed via western blotting, frequency of SMARCAL1 gene disruption analyzed via T7EI assays, and the mRNA levels of SMARCAL1 in various therapy (*n* = 3; n, no significant difference, and ^***^ *p* <0.001). g) ROS generation assays in 4T1 cells incubated with the GCZH by flow cytometry. h) Levels of SMARCAL1, cGAS, and PD‐L1 in 4T1 tumor cells after different treatments (*n* = 3; ^*^ *p* <0.05 and ^***^ *p* <0.001). The data are presented as the means ± SDs (*n* = 3). Statistical analysis was performed via Student's t test (n, no significant difference; ^*^ *p* <0.05 and ^***^ *p* <0.001).

Glucose is recognized as a pivotal energy substrate for cell proliferation, with proliferation rates directly proportional to the glucose concentration. Tumor cells are highly sensitive to fluctuations in glucose concentration, as their primary energy metabolism relies on aerobic glycolysis.^[^ ^24^ ^]^ In addition, SMARCAL1 has been implicated in various aspects of tumor biology, including migration, invasion, metastasis, and immune evasion.^[^ ^10^ ^]^ In this context, glucose‐responsive SMARCAL1 silencing via the CRISPR/Cas9 system is shown in **Figure** **4**a. The catalytic activity of GOx within GCZH increased acidity due to the side product gluconic acid, which in turn gradually induced the decomposition of GCZH. This decomposition facilitated the release of the Cas9/SMARCAL1 plasmid, thereby enhancing the silencing of the SMARCAL1 gene. First, we examined the effect of GCZH on tumor cell growth under different glucose concentrations. Under conditions without GCZH, the proliferation rates of 4T1 cells were proportional to the glucose concentration (Figure S11, Supporting Information). The cytotoxicity of GCZH increased with the addition of glucose, indicating that the cytotoxicity was glucose concentration‐dependent (Figure 4b). To determine the roles of specific components in the delivery system, a pX480 plasmid that can express GFP was encapsulated in the delivery system to assess the effect of glucose on transfection. As shown in Figure 4c, in the presence of glucose, 4T1 cells transfected with pX480‐loaded GCZH presented strong green fluorescence, whereas cells in the absence of glucose presented a weak GFP signal. Moreover, we found that the glucose concentration could modulate the degree of GFP expression (Figure 4b) because glucose oxidation enhances acidity and induces the dissociation of glucose to release plasmids, leading to increased GFP expression. Furthermore, to detect the effect of glucose on SMARCAL1 gene editing, we exposed 4T1 cells to GCZH loaded with the Cas9/sgSMARCAL1 plasmid, both with and without glucose (1 mm). Figure 4d and Figure S12 (Supporting Information) demonstrated that the expression level of SMARCAL1 in 4T1 cells treated with GCZH was reduced in the presence of glucose compared to its absence. T7 endonuclease I (T7EI) digestion analysis was used for determining the efficacy of gene editing. Figure 4e and Figure S13 (Supporting Information) illustrated that distinct T7EI digestion bands were visible and separate from the uncut band at the SMARCAL1 genome site. The indel frequency induced by the GCZH was higher in the presence of glucose than in its absence. The gene editing frequency induced by the GCZH was statistically analyzed through indel frequency assessment, Western blotting, and qPCR (Figure 4f). These results collectively revealed that the GCZH efficiently edited the SMARCAL1 gene in the presence of glucose. We subsequently investigated the effect of SMARCAL1 knockdown by GCZH on the STING signaling pathway. In addition to the modulation of SMARCAL1 expression through the CRISPR/Cas9 system of GCZH, the glucose‐responsive release of zinc ions is pivotal in activating the STING signaling pathway.^[^ ^25^ ^]^ In parallel, the GOx catalytic activity of GCZH resulted in the production of H_2_O_2_, which could be further converted into •OH by the catalytic action of Co, leading to the activation of the STING signaling pathway.^[^ ^26^ ^]^ Flow cytometry results demonstrated that GCZH significantly increased intracellular reactive oxygen species (ROS) levels in the presence of glucose, indicating its excellent ROS‐generating ability (Figure 4g). Additionally, we quantified cGAS and PD‐L1 levels via enzyme‐linked immunosorbent assay (ELISA). The data consistently revealed that in the presence of glucose, GCZH significantly increased cGAS levels and decreased PD‐L1 levels compared with those in the control groups (Figure 4h), indicating that compared with control conditions, the GCZH NPs effectively activate the STING pathway and block PD‐L1‐mediated immune checkpoint inhibition in the presence of glucose.

### 2.4 In Vitro Antitumor Activity of GCZH

**Figure 5:**
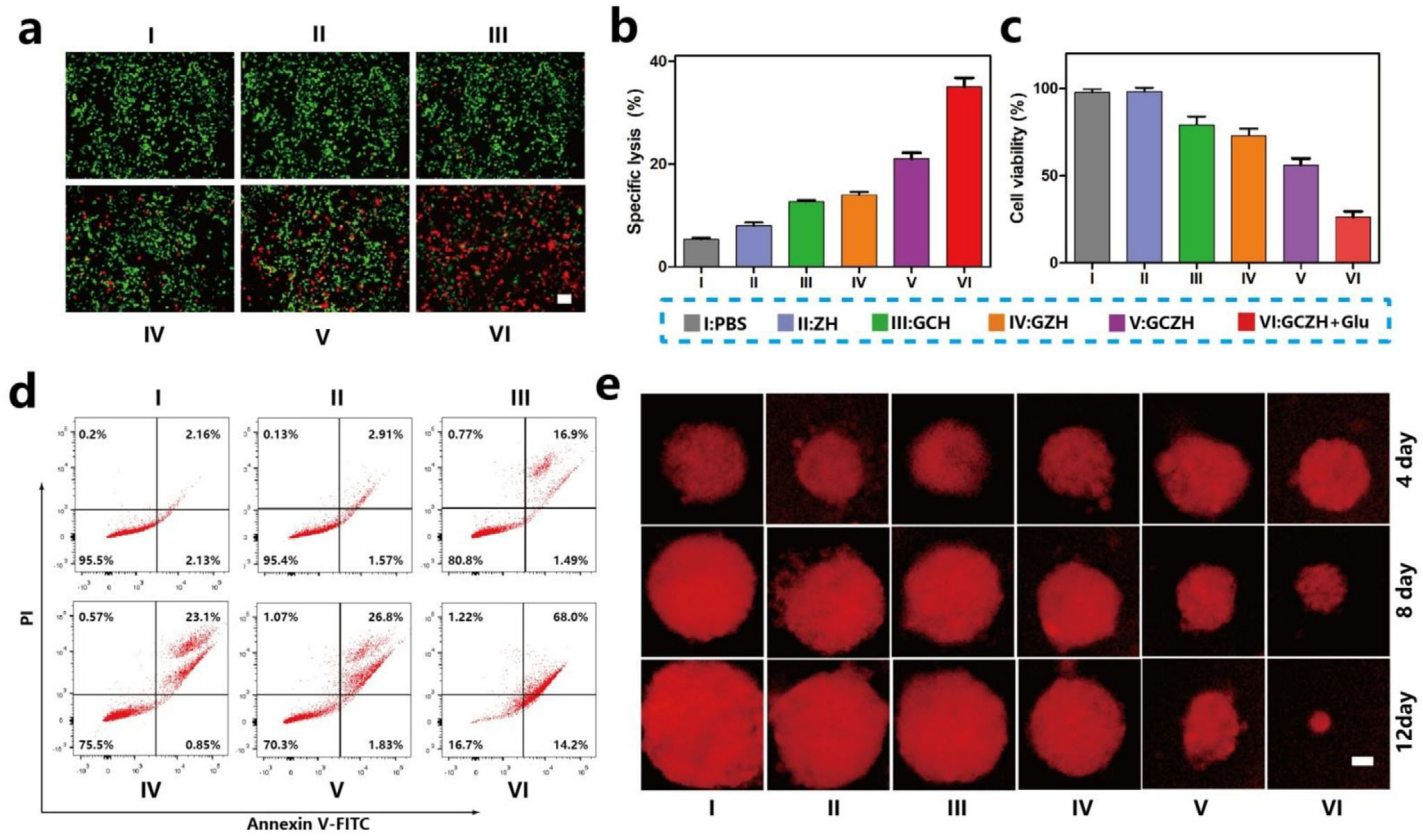
In vitro antitumor activity of GCZH. a) Calcein‐AM&PI staining for identifying the live or dead 4T1 cells in different groups. Scale bar: 30 µm. b,c) Specific lysis (b), and cell viability (c) of 4T1 cells in different groups (mean ± SD, *n* = 3). d) Flow cytometry showing the results of the apoptosis assay based on Annexin V‐FITC and propidium iodide staining of 4T1 cells in different groups. e) Photographs and volume of MCTS on different days for various groups, including the control. Scale bar: 50 µm. The data are presented as the means ± SDs (*n* = 3). Statistical analysis was performed via Student's t test (n, no significant difference; ^*^ *p* <0.05 and ^***^ *p* <0.001).

The glucose oxidation catalyzed by GOx was found to be pivotal in activating and potentiating glucose‐dependent cytotoxicity.^[^ ^27^ ^]^ Thus, we subsequently investigated the targeting efficacy of GCZH toward cancer cells via fluorescence microscopy. For this purpose, rhodamine B (RhB)‐loaded GCZH particles were used to evaluate the cellular internalization efficiency. The fluorescence images demonstrated that the GCZH NPs began to accumulate in the 4T1 tumor cells within 4 h (Figure S14, Supporting Information), suggesting that GCZH possesses a high targeting capacity for cancer cells. Subsequently, 4T1 cells were subjected to various treatments and stained with a live–dead cell staining kit for examination via confocal laser scanning microscopy (CLSM). Notably, compared with the other treatment groups, the cells treated with GCZH (Glu) presented significant red fluorescence, which is indicative of cell death (**Figure** **5**a). In contrast, cells treated with GCZH displayed diminished red fluorescence, and no distinct red fluorescence was observed in the GZH and GCH groups. The cytotoxicity of the NPs was further assessed via lactate dehydrogenase (LDH) and methylthiazolyl tetrazolium (MTT) assays. As illustrated in Figure 5b,c, the GCZH (Glu) group demonstrated exceptional cytotoxicity against 4T1 cells, which exhibited GCZH concentration‐dependent cytotoxicity (Figure S15, Supporting Information). Specifically, the tumor cell death caused by GCZH was attributed to the generation of ROS caused by the consumption of glucose and the generation of highly toxic •OH via the Fenton reaction. Upon staining the cells with Annexin V‐FITC and propidium iodide (PI), we determined that ≈68.0% of the GCZH (Glu)‐treated cells underwent late apoptosis, whereas the percentages of late apoptotic cells were 26.8% and 23.1% following the GCZH and GZH treatments, respectively (Figure 5d). Consequently, to precisely assess the effect of GCZH on tumor cells under glucose conditions, we employed multicellular tumor spheroids (MCTSs). As 3D tumor models, MCTSs have become a staple in cancer therapeutics because of their ability to replicate the acidic and enzymatic microenvironments characteristic of solid tumors.^[^ ^28^ ^]^ In this study, MCTSs were generated through the cultivation of 4T1 tumor cells. After four days in culture, the MCTSs reached an approximate diameter of 200 µm, at which point they were exposed to treatments with different nanomaterials. A set of MCTSs that received PBS treatment served as the control group. The volume of spheroids across all groups was monitored over various days. As depicted in Figure 5e, the volumes of 3D MCTSs in both the PBS and ZH groups gradually increased, whereas the introduction of GCZH and GCZH (Glu) notably suppressed the growth of 3D MCTSs. After the addition of GCZH under glucose conditions, the majority of the tumor cells were eradicated by day 12. Figure 5e shows that by day 12, the 3D MCTS volumes had expanded 3.5‐fold in the PBS group and 2.2‐fold in the ZH group compared with their sizes on day 4. In stark contrast, the volume of 3D MCTSs in the GCZH (Glu) group decreased by 90.1%, further corroborating the superior cytotoxic efficacy of GCZH (Glu) against tumor cells across all the tested groups. All of these data demonstrate that GCZH NPs with glucose have potential applications in cancer therapy.

### 2.5 GCZH Induces Potent Immune Responses

**Figure 6:**
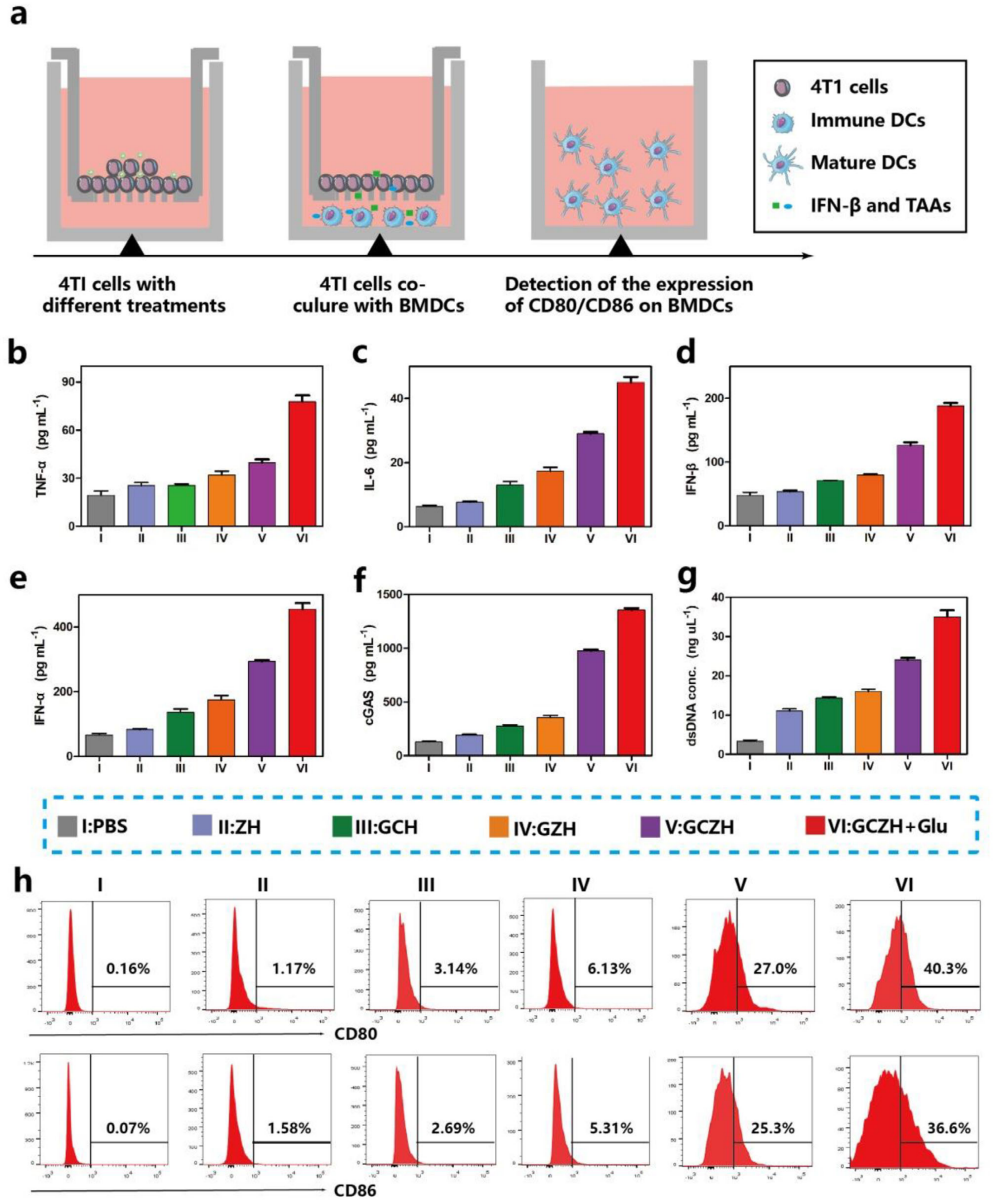
In vitro ability of GCZH to activate DCs. a) Schematic of the experimental design using the Transwell system. The upper compartment was filled with 4T1 cancer cells, and the bottom chamber was cultured with BMDCs. b–e) TNF‐α (b), IL‐6 (c), IFN‐𝛽 (d), and IFN‐α (e) concentrations in the supernatant of BMDCs after treatment with 4T1 tumor cells treated with different formulations (mean ± SD, *n* = 3). f,g) Concentrations of cGAS (f) and dsDNA (g) in the supernatants of 4T1 cells after treatment in different groups (means ± SD, *n* = 3). h) Representative flow cytometric analysis of the expression levels of CD86 and CD80 after incubation of BMDCs with the supernatant of 4T1 tumor cells treated with different formulations.

Given the favorable cascade‐activating anticancer results, we investigated the immunostimulatory effects of GCZH using bone marrow‐derived dendritic cells (BMDCs) isolated from 6–8‐week‐old BALB/c mice. We subsequently investigated the ability of NP‐treated 4T1 tumor cells to activate DCs. Using a Transwell system, we conducted coculture experiments with DCs and GCZH‐treated 4T1 cells (**Figure** **6**a). We quantified the secretion levels of immunostimulatory cytokines via ELISA. The GCZH (Glu)‐treated group presented significant increases in the levels of tumor necrosis factor α (TNF‐α), interleukin 6 (IL‐6), interferon β (INF‐β), and interferon α (INF‐α) (Figure 6b–e), indicating that in situ GCZH (Glu) could induce the secretion of cytokines by BMDCs,^[^ ^29^ ^]^ which was attributed to the activation of the STING signaling pathway by the GCZH NPs. We subsequently investigated the effect of GCZH on the STING signaling pathway. The results consistently demonstrated that, compared with the control, GCZH and GCZH (Glu) induced significant increases in the concentrations of cGAS and IFN‐α/β (Figure 6d–f). IFN is a hallmark cytokine of STING pathway activation, and it plays a crucial role in the activation of subsequent innate immunity and the connection of adaptive immunity.^[^ ^30^ ^]^ As shown in Figure 6g, the dsDNA concentration significantly increased in cells treated with GCZH and GCZH (Glu) compared with the control group. The dsDNA helps to increase cGAS/STING signal transduction and generate a large amount of ROS to damage mitochondria and induce tumor cell death. The release of HMGB1 (a marker of immunogenic cell death) from cells treated with GCZH and GCZH (Glu) was, respectively, 3.57 times and 6.62 times greater than that of the control cells (Figure S16, Supporting Information), which was beneficial for the maturation and differentiation of DC cells.^[^ ^31^ ^]^ Flow cytometry was used to examine the expression of CD80 and CD86 (Figure 6h). The results demonstrated significant upregulation of the costimulatory ligands CD80 and CD86 in the GCZH (Glu) groups, with 40.3% CD86 and 36.6% CD80 expression, respectively. These results suggest that the glucose‐response CRISPR vaccine effectively induces DC maturation, indicating its substantial potential for enhancing antitumor therapy.

### 2.6 In Vivo Antitumor Effect of GCZH

Upon thorough characterization of the properties of GCZH, we proceeded to assess the in vitro cytotoxicity of ZnCo‐ZIF, GCZ, and GCZH against NIH 3T3 mouse embryonic fibroblasts via the MTT assay. As depicted in Figure S17 (Supporting Information), no significant cytotoxic effects were observed for ZnCo‐ZIF, GCZ, or GCZH on these normal cells after either 24 or 48 h of coincubation. The results revealed that ZnCo‐ZIF was endotoxin‐free and suitable for subsequent biological applications. We then designed a subcutaneous murine 4T1 tumor model to evaluate the therapeutic effects of the ZnCo‐ZIF NPs in vivo. First, we thoroughly assessed the in vivo toxicity of GCZH to ensure its safe bioapplication. We performed hematological analysis and blood biochemical tests on the GCZH NPs. A hemolysis rate of ≈4% was observed when the concentration of GCZH was 400 µg mL^−1^ (Figure S18, Supporting Information). The low hemolysis rate indicated that GCZH was safe for injection. Liver and kidney function parameters remained unchanged between the control and GCZH‐treated groups at various time points (Figure S19, Supporting Information), indicating that infection and inflammation were insignificant throughout the experiments. Compared with the control group, the injection of GCZH had little influence on mouse growth (Figure S20, Supporting Information). Furthermore, owing to the high histocompatibility and favorable biocompatibility of the GCZH NPs, their effects in major organs were obtained and evaluated to clarify their safe bioapplications (Figure S21, Supporting Information). Hematoxylin and eosin (H&E) staining of the main organs did not significantly differ among different groups (Figure S22, Supporting Information), further illustrating the favorable biocompatibility of the biomimetic nanocatalysts.

**Figure 7:**
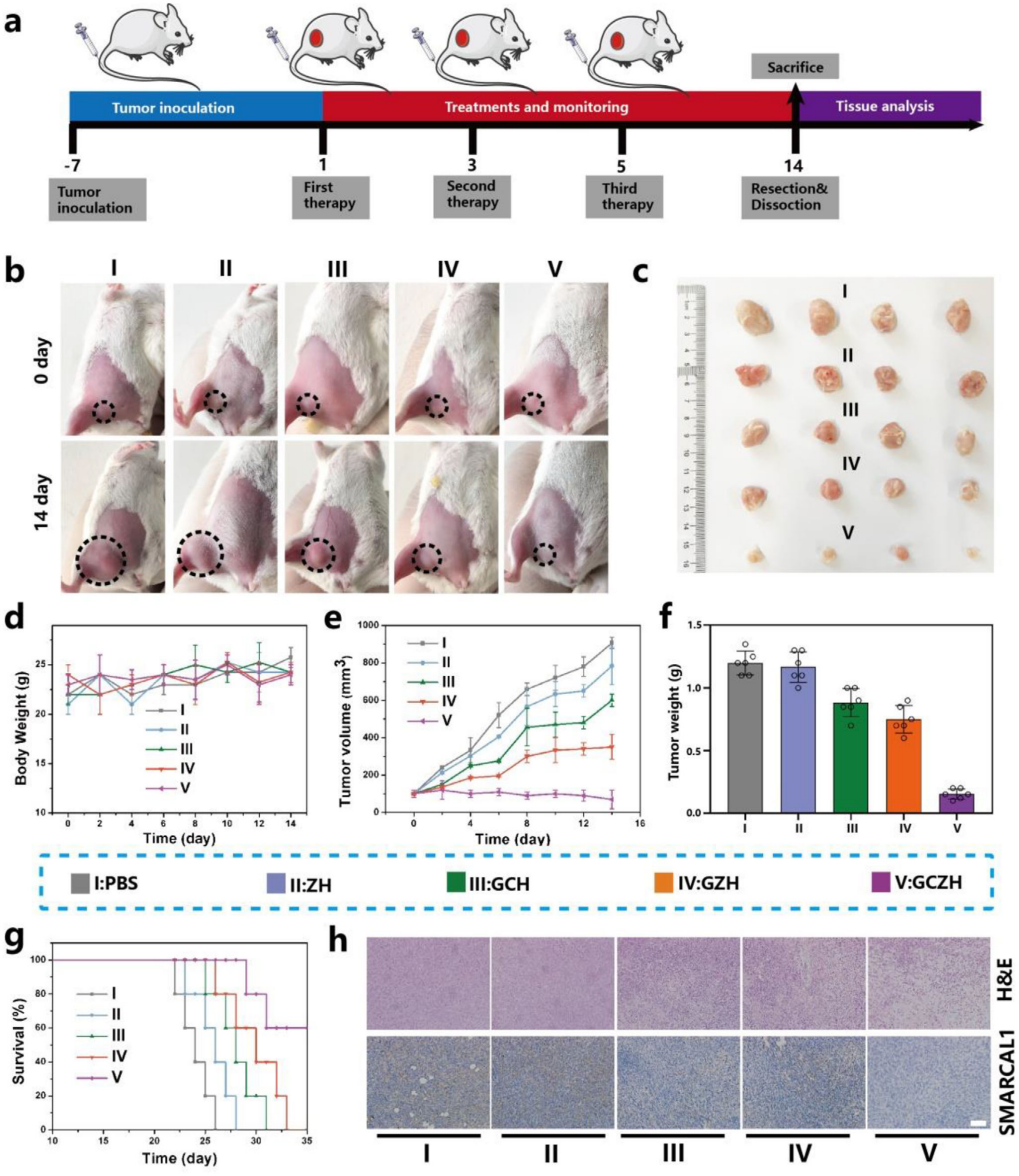
Antitumor efficacy of the GCZH in 4T1 tumor‐bearing mice. a) Schematic representation of 4T1 tumor xenograft establishment and the therapeutic process. b) Photographs of tumor‐bearing mice prior to treatment and following 14 days of various treatments. c) Digital photographs of dissected tumors of mice in different treatment groups. d–f) Body weights (d), tumor volume changes (e), and tumor weights (f) in different treatment groups (mean ± SD, *n* = 6). g) Survival of 4T1 tumor‐bearing mice after different treatments. h) H&E staining and immunofluorescence images of SMARCAL1 in tumor tissue after 14 days of treatment. The scale bar is 100 µm.

Next, we evaluated the anticancer properties of GCZH in tumor‐bearing mice. 4T1 breast tumors were injected subcutaneously into BALB/c mice to establish a tumor‐bearing mouse model. After systemic administration, we assessed the biodistribution and accumulation of GCZH at the tumor site. As shown in Figure S23 (Supporting Information), upon surgical removal and imaging of the main organs and tumors, the Cy3‐loaded GCZH‐treated samples presented stronger fluorescence than the control samples did, primarily due to HA, which enhanced blood circulation and tumor accumulation of GCZH (Figure S24, Supporting Information). The establishment of 4T1 tumor xenografts and the therapeutic process are shown in **Figure** **7**a. Mice with tumors ≈100 mm^3^ in size were randomly divided into five groups and administered PBS, ZH, GCH, GZH, or GCZH. Tumor volume and mouse body weight were measured for 14 days from the initial administration. The results showed that the GCH and GZH treatments suppressed tumor growth to some extent, whereas the GCZH treatment resulted in the most significant inhibition of tumor growth (Figure 7b,c). As shown in Figure 7d, different treatments had little influence on mouse growth. The tumor volumes (Figure 7e) of the treatment groups were consistent with the measured weights (Figure 7f) after 14 days of therapy. The survival rates of the treatment groups further demonstrated that the GCZH group presented the best antitumor efficacy. Owing to effective tumor suppression, the survival rate of the mice treated with GCZH improved to ≈60% after 35 days (Figure 7c). H&E staining and SMARCAL1 immunohistochemistry assays were performed to further analyze the therapeutic efficacy of various treatments. H&E staining of the tumor histopathology revealed that GCZH could induce cancer cell apoptosis (Figure 7h). Consistent with the tumor volume results, mice treated with GCZH presented the most prominent necrosis and decreased SMARCAL1 expression, suggesting excellent tumor inhibition efficacy (Figure 7h). Notably, the significantly greater extent of tumor inhibition in the GCZH group than in the other groups indicated that SMARCAL1 blockade could significantly improve therapeutic efficacy. These remarkable therapeutic benefits are attributed to the ability of GCZH to effectively accumulate at tumor sites and inhibit tumor development.

### 2.7 GCZH‐Induced In Vivo Antitumor Immune Responses

**Figure 8:**
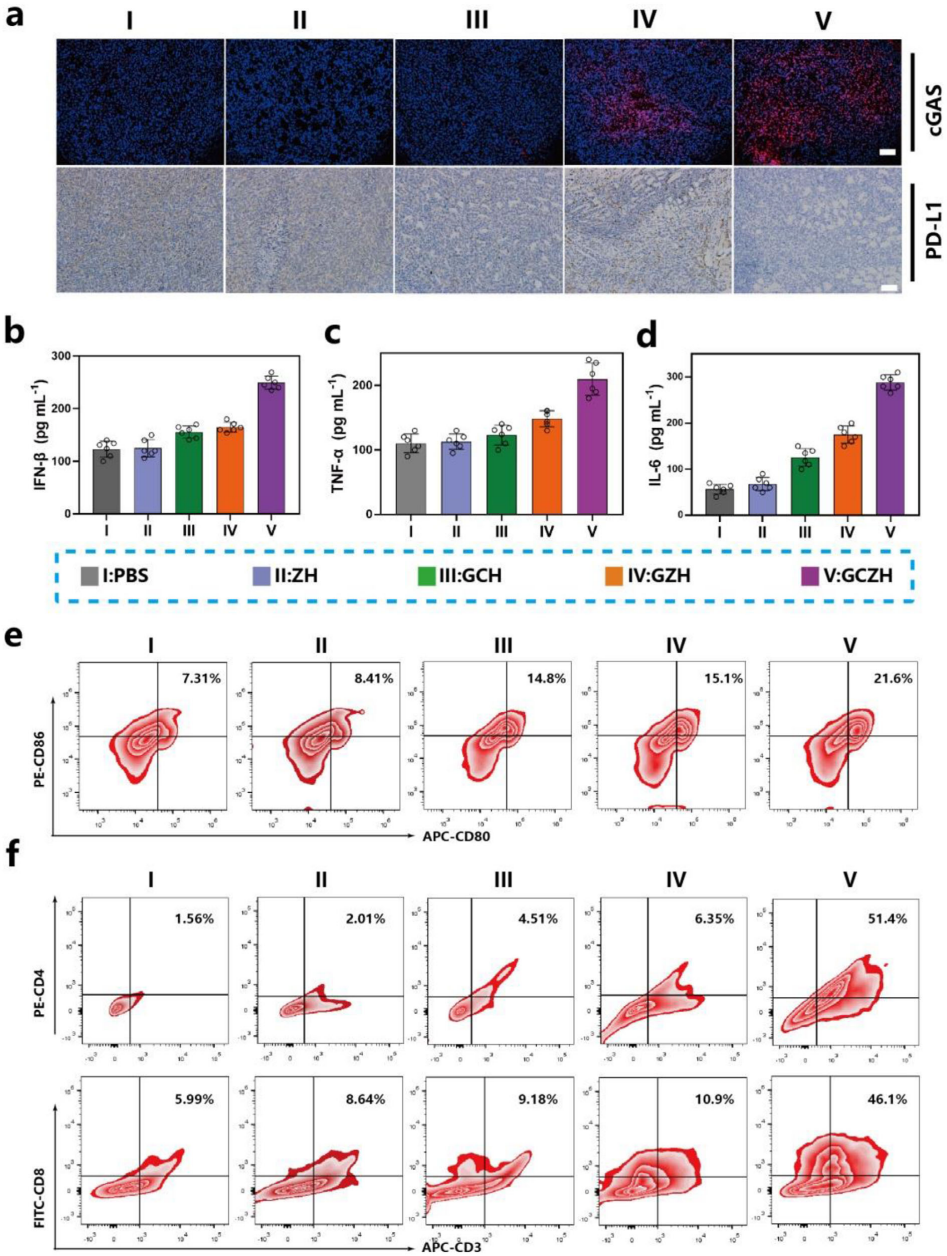
Maturation of DCs and the T‐cell‐mediated antitumor immune response. a) Immunostaining images of cGAS and PD‐L1 in tumor sections from different treatment groups. Scale bar: 100 µm. b–d) IFN‐𝛽 (b), TNF‐𝛼 (c), and IL‐6 (d) were measured by ELISA (mean ± SD, *n* = 6). e) Representative flow cytometric analysis and quantification of mature DC (D80^+^ and D86^+^) proportions in different tumor tissues. f) Flow cytometric analysis of the percentages of CD4^+^ T cells and CD8^+^ T cells after different treatments.

To elucidate the antitumor mechanism of the GCZH NPs, we analyzed tumor tissue sections from different treatment groups through histopathological immunohistochemistry and immunofluorescence. Treatment with GCZH decreased PD‐L1 expression and increased cGAS expression, further confirming the activation of the STING pathway and PD‐L1‐mediated immune checkpoint inhibition (**Figure** **8**a). The levels of the tumor cytokines IFN‐𝛽, TNF‐𝛼, and IL‐6 were quantified via ELISA kits. As shown in Figure 8b–d, compared with those in the PBS group, the secretion levels of IFN‐𝛽, TNF‐𝛼, and IL‐6 in the GCH and GZH groups significantly increased, and these increases were more pronounced to those in the PBS group. To further elucidate the antitumor immune response induced by GCZH, we analyzed the infiltration levels of antitumor immune cells within tumors via flow cytometry. Given the pivotal role of DC maturation in triggering the T‐cell immune response,^[^ ^32^ ^]^ we investigated the maturation of DCs (CD80^+^ and CD86^+^) in the lymph nodes of mice receiving different treatments. The percentage of DCs (CD80^+^ and CD86^+^) significantly increased to 21.6% in GCZH‐treated mice, which was 2.9‐ and 2.5‐fold greater than that in the PBS and ZH groups, respectively, indicating efficient DC stimulation by GCZH (Figure 8e). The percentage of effector T cells infiltrating tumors was then analyzed. There are two main types of effector T cells: CD8^+^ T cells and helper T lymphocytes (CD4^+^ T cells).^[^ ^33^ ^]^ CD8^+^ T cells can kill cancer cells directly, whereas CD4^+^ T cells regulate other immune cells. GCZH‐treated tumors presented increased CD4^+^ T and CD8^+^ T cells in tumor tissues. As shown in Figure 8f, the proportions of CD8^+^ T and CD4^+^ T cells in the GCZH‐treated group increased markedly to 46.1% and 51.4%, respectively, nearly ten times higher than those in the PBS group, indicating that GCZH enhanced infiltration of CD4^+^ T and CD8^+^ T cells into tumor tissues. These findings demonstrate that GCZH can effectively boost antitumor immune responses that inhibit tumor development.

## 3 Conclusion

We described a glucose‐responsive CRISPR‐based nanovaccination with SMARCAL1 blockade to enhance STING signaling and inhibit the opposing functions of interferons, thereby suppressing tumor growth. Nanovaccination, with Fenton reaction activity and glucose consumption ability, can produce ROS and endogenous dsDNA, effectively activating STING. Gene silencing of SMARCAL1 enhances endogenous dsDNA damage to improve cGAS‐STING‐dependent IFN secretion. Moreover, PD‐L1 expression is inhibited in cancer cells, resulting in the termination of PD‐L1‐mediated opposing functions of interferon. Our study provides a convenient strategy for enhancing STING signaling and inhibiting type I interferon‐mediated immunosuppressive feedback. The functionalized genome‐editing system and the glucose‐targeted therapeutic paradigm will provide significant directions in nanotechnology.

## 4 Experimental Section

### Reagents, Materials, and Mice

Cobalt chloride hexahydrate (CoCl_2_•6H_2_O) and zinc nitrate hexahydrate (Zn(NO_3_)_2_•6H_2_O) were obtained from Xilong Chemicals (Guangdong, China). 2‐Methylimidazole (2‐mim) and hyaluronic acid were obtained from Aladdin Reagent (Shanghai, China). H_2_O_2_ (w/w: 30%) solution was purchased from Sinopharm Chemical Reagent Co., Ltd. (China). Rhodamine B (RhB) was procured from Sigma–Aldrich. MTT was purchased from Amresco. Cy3 was acquired from Macklin Reagent (Shanghai, China). Glucose oxidase was obtained from Shanghai Yuanye Biotechnology Co., Ltd. (Shanghai, China). Lactate dehydrogenase (LDH) release assay kits, an HMGB1 enzyme‐linked immunosorbent assay (ELISA) kit, and a BCA protein assay kit were brought from Beyotime Institute of Biotechnology (China). A hydrogen peroxide (H_2_O_2_) colorimetric assay kit was obtained from Solarbio (Beijing, China). ELISA kits for cGAS, PD‐L1, TNF‐α, IFN‐α, IFN‐β, and IL‐6 were obtained from Energy Chemical. A 75 µm nylon cell strainer was purchased from Corning. T7 endonuclease I was obtained from New England Biolabs. The pX458 and pX330 plasmids were purchased from Invitrogen. All other reagents were of analytical grade. In accordance with a previous report, a CRISPR/Cas9 plasmid targeting the SMARCAL1 gene was designed via the online CRISPR Design Tool (http://crispr.mit.edu/), and a sgRNA with the target guide sequence GCTCAGAGAGTGTAACGCCC was found to have satisfactory efficiency in downregulating SMARCAL1.

Healthy female BALB/c mice (20–25 g) were purchased from Cavens Laboratory Animal Co., Ltd (Changzhou, China), and all experiment procedures were according to the guidelines of the Ethical Committee of Hainan Medical University for animal experiments (approval number: No. HYLL‐2024‐541).

### Preparation of GCZH

2‐Methylimidazole (500 µL, 1.1 g mL^−1^), GOx (5 µg), plasmids (100 ng), and CoCl_2_•6H_2_O (150 µL, 300 mg mL^−1^) were dissolved in water and mixed at room temperature for 15 min. The products were then centrifuged and washed three times with water. To encapsulate hyaluronic acid (HA), 20 mg of NPs was added to 40 mL of deionized water containing 40 mg of HA and stirred at room temperature for 24 h. The product was washed with UP water three times and then freeze‐dried overnight to obtain the NPs. The GOx encapsulation efficiency (percent) was calculated by measuring protein concentrations. The loading capacity of the plasmid was investigated via UV–vis absorbance and agarose gel electrophoresis. The encapsulation efficiency was calculated via the following equation:

(1) $$\mathrm{Encapsulation}\;\mathrm{efficiency}\;=\left(W_{T}-W_{F}\right)/W_{T}\times 100\%$$

where W_T_ represents the total weight, and W_F_ denotes the weight of the unencapsulated free LOx or plasmid.

### Stability of GCZH

The GCZH NPs were dispersed in PBS at pH 7.4 and stored at 4 °C to examine their colloidal stability. The samples were periodically collected over 1 month, and the particle size and zeta potential were measured. All the experiments were performed in triplicate, and the data are presented as the means ± SDs (*n* = 3).

### Glucose Consumption Assay

The glucose consumption effect of GOx and GCZH nanoparticles was studied. A mixture of 1 mL of the GCZH NP mixture (100 µg mL^−1^) and 4 mL of the glucose mixture (5 mg mL^−1^) was shaken at 37 °C in capped vials, and the pH variation was monitored via a pH meter. Samples were collected at preset time intervals for glucose quantification via a glucose assay kit. All the experiments were performed in triplicate, and the data are presented as the means ± SDs (*n* = 3).

### H_2_O_2_ Generation Assay

H_2_O_2_ concentrations were measured via a hydrogen peroxide assay kit. A standard curve for H_2_O_2_ was generated by mixing 50 µL of H_2_O_2_ solutions with different concentrations with 100 µL of test solution and incubating at room temperature for 30 min. The absorbance at 560 nm was measured via a plate reader (BioTek Instruments Inc., USA). The concentrations of H_2_O_2_ in the treatment groups were also detected via the same procedures. All the experiments were performed in triplicate, and the data are presented as the means ± SDs (n = 3).

### O_2_ Consumption Assay

The O_2_ consumption effect of the GCZH NPs was assessed with a dissolved oxygen meter. In detail, 1 mL of the GCZH NP mixture (100 µg mL^−1^) was added to 4 mL of the glucose mixture (5 mg mL^−1^). O_2_ generation was monitored at different reaction times in an environment sealed with liquid paraffin via a dissolved oxygen meter (JPB‐607A). All of these experiments were performed in triplicate.

### Peroxidase‐Like Activity Assay

The peroxidase‐like activity of ZnCo‐MOF and GCZH was assessed using H_2_O_2_ or glucose as the substrate in the presence of TMB in pH 5.5 buffers. The absorbance of oxTMB was recorded at 300–800 nm via a JASCO V‐550 UV–vis spectrophotometer. Experiments were conducted in 100 µL of PBS (pH 5.5) containing 100 µg mL^−1^ ZnCo‐MOF, 832 µm TMB, various concentrations of H_2_O_2_, 100 µg mL^−1^ GCZH, 832 µm TMB, and different concentrations of glucose. All experiments were performed in triplicate.

### Cobalt, Zinc, and Plasmid Release Experiments

The plasmid release experiments were conducted in four groups: 1) pH 7.4; 2) pH 5.0; 3) pH 7.4 + glucose (5 mg mL^−1^); and 4) pH 5.0 + glucose (5 mg mL^−1^) phosphate‐buffered saline (10 mm). Cobalt and zinc release experiments were performed in two groups with different pH values (pH 7.4 and 5.0). GCZH (1 mg mL^−1^, 5 mL) was prepared in 5 mL of PBS corresponding to each group, and the mixtures were stirred at 37 °C in capped vials. At predetermined time intervals, a portion of the buffer solution was removed and centrifuged for UV–vis spectrum measurement of the plasmid or ICP‒MS measurement of cobalt and zinc. The experiments were all performed in triplicate, and the data are presented as the means ± SDs (*n* = 3).

### Effect of Glucose on Plasmid Expression

4T1 cells were seeded in a 24‐well plate at a density of 1 × 10^5^ cells per well and then incubated at 37 °C for 24 h. pX458‐loaded GCZH (50 µg mL^−1^) was added. After 4 h, the cells were co‐incubated with different concentrations of glucose at 37 °C for 48 h. GFP expression was visualized via fluorescence microscopy.

### Gene Disruption Assay

4T1 cells were seeded in the well of a 6‐well plate at a density of 2 × 10^5^ cells per well and incubated at 37 °C for 24 h. The medium was subsequently replaced with new culture medium containing a particular agent (50 µg mL^−1^ GCZH with or without 1 mm glucose), and the cells were cultured at 37 °C for 48 h. Untreated cells were also studied for comparison. The cells were harvested for T7E1, western blot, and qPCR assays. T7E1 assays: the genomic DNA was extracted and transcribed via two specific PCR primers (5′‐TCAGGTTCACACCCTTTGCTAACCCAA‐3′ and 5′‐TCTTAAACACTGCAATGAGTTCCGCAT‐3′) located near the target gene to acquire the PCR products. The following PCR program was used: 95 °C for 3 min; 95 °C for 30 s; 50 °C for 30 s; 72 °C for 30 s for 35 cycles; and 72 °C for 5 min. After purification and quantification, the PCR products were digested with T7 endonuclease I (T7E1) to determine the disruption efficiency of the SMARCAL1 gene. The digested DNA was then analyzed via 2% agarose gel electrophoresis and imaged via a gel documentation system. Indel formation efficiencies were calculated via ImageJ. qPCR assays: the sequences of the qPCR primers used were as follows: SMARCAL1 forward primer: 5′‐ATAGTGCCCTGATGAAAGCA‐3′; SMARCAL1 reverse primer: 5′‐ATGTCTGCCTCGAAGTAGGC‐3′; GAPDH forward primer: 5′‐AGCATGCAAACCTTCCCTGA‐3′; and GAPDH reverse primer: 5′‐ CTGGGTTATTTCCCTGGCGT‐3′. All the experiments were performed in triplicate. The data are presented as the mean values ± SDs (*n* = 3).

### Assessment of Intracellular ROS Levels

To detect ROS, treated cells were washed twice with PBS and subsequently incubated with 10 µM DCFH‐DA for 30 min. Following the removal of the unloaded probe with PBS, the fluorescence intensity of the cells was measured via flow cytometry.

### Cell Viability Assay and In Vitro Cytotoxicity

The in vitro cytotoxicities of the NPs were evaluated via an MTT assay. 4T1 cells were seeded into 96‐well microtiter plates and incubated overnight to allow adherence. The cells were incubated with NPs at the indicated concentrations for 24 h under specific conditions. Each well received 20 µL of MTT solution. Cell viability was measured using a microplate spectrophotometer. The data are reported as the means ± SDs (*n* = 3).

### LDH Release Assay

4T1 cells were seeded in 24‐well plates at a density of 1 × 10^5^ cells mL^−1^ and incubated for 24 h. Different NPs were added and cocultured for 48 h. The released LDH in supernatant was measured according to the manufacturer's instructions. The specific lysis of 4T1 cells was calculated as follows: (OD (4T1 cells + GCZH)‐OD (4T1 cells without GCZH))/OD (4T1 cells+LDH release reagent) × 100%, where the OD represents the absorbance of the LDH testing solution at 490 nm. All the experiments were performed in triplicate, and the data are presented as the means ± SDs (*n* = 3).

### Annexin V‐FITC/PI Assay

To investigate the mechanisms of death induced by various NPs, apoptosis assays were performed on 4T1 cells. The cells were plated in 24‐well plates at a density of 1 × 10^5^ cells per well and allowed to adhere overnight. The cells were subsequently coincubated with different NPs for 12 h. Following the manufacturer's instructions, the cells were stained with Annexin V‐FITC/PI (Beyotime, China) and analyzed via flow cytometry.

### Live/Dead Assay

To assess cell viability, 4T1 cells were seeded in glass‐bottom dishes at a density of 1 × 10^5^ cells per well. After 24 h of treatment with different NPs, cell viability was evaluated via a live/dead kit (BestBio, China). The dyes Calcein AM and propidium iodide (PI) from the kit were used to stain live and dead cells, respectively. Fluorescence microscopy was used to observe the fluorescence of calcein AM and PI.

### MCTS Experiments

3D MCTSs were established by seeding 1.0 × 10^3^ 4T1 cells per well into an agarose precoated 96‐well plate and incubating at 37 °C in a humidified atmosphere with 5% CO_2_ for MCTS formation. After 4 days of growth, the MCTSs were treated with different NPs (100 µg mL^−1^). Half of the old culture medium was replaced with an equal amount of fresh culture medium daily. The diameters of the MCTSs in different groups were recorded every 4 days.

### Extracellular dsDNA and HMGB1 Detection Assay

4T1 cells were seeded in six‐well plates at a density of 1 × 10^5^ cells mL^−1^ and incubated for 24 h. Different NPs were added and cocultured for 48 h. The released dsDNA in the supernatant was measured with a NanoDrop 2000c Spectrophotometer. HMGB1 release was measured with an HMGB1 ELISA Kit. All the experiments were performed in triplicate, and the data are presented as the means ± SDs (*n* = 3).

### BMDC Maturation In Vitro

4T1 cells that had been pretreated with either PBS or NPs for 24 h were added to the upper layer of the Transwell chambers (0.4 µm pores, Corning) and coincubated with the BMDCs for an additional 24 h. Subsequently, the DCs were incubated with the appropriate antibodies. The stained cells were then subjected to flow cytometry for analysis. The supernatants of treated BMDCs were collected, and the cytokine contents were measured via a mouse ELISA kit. All the experiments were performed in triplicate, and the data are presented as the means ± SDs (*n* = 3).

### Hemolysis Test of GCZH

One milliliter of whole blood was drawn from the orbital venous plexus of each mouse into a tube containing Li‐heparin. The blood was mixed with a proper volume of 10 mm PBS (pH = 7.4) and centrifuged at 2500 rpm for 5 min, after which the supernatant was discarded. This washing process was repeated three to four times until the supernatant was clear and transparent. The precipitated erythrocytes were then dispersed in 10 mm PBS (pH = 7.4) to get an erythrocyte suspension. The GCZH NPs were added to the erythrocyte suspension and then were incubated for 8 h at room temperature. The specific 540 nm spectrophotometric absorptions of hemoglobin were measured. All the experiments were conducted in triplicate, and the data are presented as the means ± SDs (*n* = 3).

### In Vivo Biocompatibility of the GCZH Nanoparticles

Thirty healthy female mice were randomly assigned to three groups and received intravenous injections of the GCZH NPs (5 mg kg^−1^, 200 µL). To assess in vivo biosafety, the body weights of the mice were monitored every 4 days. At the indicated time, the mice were euthanized, and blood samples were collected for comprehensive blood panel analysis and serum biochemistry tests at the Third Affiliated Hospital of Shenzhen University. Additionally, major organs (heart, liver, spleen, lung, and kidney) were harvested, fixed in 10% paraformaldehyde, processed into paraffin, sectioned to ≈4 µm, and stained with H&E.

### Tumor Model and In Vivo GCZH Treatment

For the tumor model, 4T1 cells (1 × 10^6^) suspended in PBS were injected subcutaneously into the inner thigh of each female BALB/c mouse. Once the tumor volume reached ≈100 mm^3^, the mice were weighed and randomly assigned to five groups, with six mice per group. The mice were then administered 50 µL of intravenous injections of PBS, ZH, GCH, GZH, or GCZH (5 mg kg^−1^). Tumor growth was subsequently monitored by measuring the tumor volume.

### In Vivo Biodistribution of GCZH

In the biodistribution analysis, 4T1 tumor‐bearing mice were intravenously injected with Cy3‐labeled GCZH (50 µL, 5 mg kg^−1^). At 12 h post administration, the mice were sacrificed, and the tumor tissues and major organs, including the heart, liver, spleen, lung, and kidney, were harvested for fluorescence imaging.

### Flow Cytometry Assay in Tumor Tissue

After therapy, the mice were sacrificed, and the tumor tissues were obtained and digested into single‐cell suspensions. The cell suspension was filtered through a 70 µm nylon strainer, centrifuged at 400 g for 5 min, washed with PBS containing 2% FBS, and then treated to block nonspecific protein binding. The appropriate antibodies were added at the recommended dosages and incubated at 4 °C for 30 min. The cells were subsequently washed twice with PBS, resuspended in 500 µL of PBS, and analyzed via flow cytometry.

### H&E, Immunohistochemistry, and Immunofluorescence Staining of Tumor Tissue

For histological analysis, major organs and tumors were excised from the mice following euthanasia. The collected tumors and organs were fixed in 10% paraformaldehyde, embedded in paraffin, sectioned into ≈4‐µm thick slices, and stained with H&E. To determine the intratumoral status, paraffin‐embedded sections of 4T1 tumor tissues were deparaffinized, hydrated, and subjected to antigen retrieval for immunohistochemistry and immunofluorescence staining against SMARCAL1, cGAS, and PD‐L1.

### Statistical Analysis

All figures presented in this article were obtained from at least three independent experiments (n ≥ 3) that yielded consistent results. Sample data were all presented by mean value ± standard deviations (SDs). Outliers were identified using the Grubbs' test and excluded if they exceeded the threshold of *p* <0.05. Kaplan‐Meier curves were used to analyze the mouse survival benefit. Statistical differences were evaluated via unpaired Student's two‐sided t tests with GraphPad Prism 8.0 (GraphPad Software). A *p* value <0.05 was considered to indicate a significant difference between the data (^*^ *p* <0.05, ^**^ *p* <0.01, and ^***^ *p* <0.001; n, no significant difference).

## Conflict of Interest

The authors declare no conflict of interest.

## Supporting information



Supporting Information

## Data Availability

The data that support the findings of this study are available in the supplementary material of this article.
