# Correction to Advanced Cancer Immunotherapy via SMARCAL1 Blockade Using a Glucose‐Responsive CRISPR Nanovaccine

**DOI:** 10.1002/advs.202520638

**Published:** 2025-11-05

Y. Li, Y. Zhang, C. Li, G. Chen, P. Muhammad, Y. Yao, L. Gao, Z. Liu, Y. Wang, Advanced Cancer Immunotherapy via SMARCAL1 Blockade Using a Glucose‐Responsive CRISPR Nanovaccine. *Advanced Science* **2025**, *12* (37), e02929. https://doi.org/10.1002/advs.202502929

Concerns were raised by a third party regarding duplication between the third graph and the sixth graph of Figure 4c; however, these graphs depict the same experimental results (the same experimental conditions: GCZH with 1.0 mm glucose).

In Figure 5a, the first and second graphs were mistakenly duplicated, and the third and fourth graphs were incorrect. The corrected figure is shown below. This correction does not affect the overall findings and conclusions of this paper.

Corrected Figure 5a


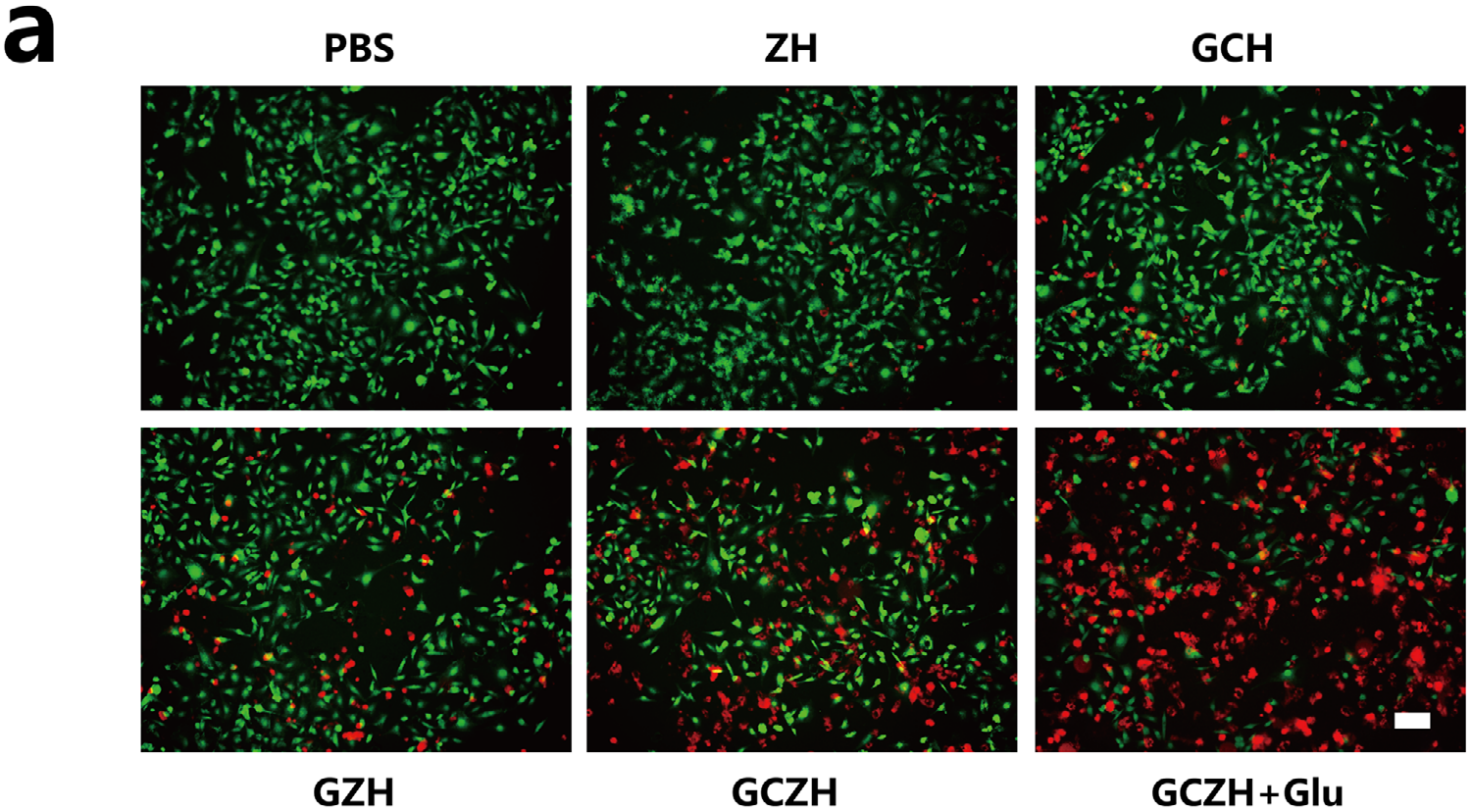


In Figure 8a, the immunohistochemistry image of PD‐L1 in the PBS group was incorrect. The revised figure is provided below.


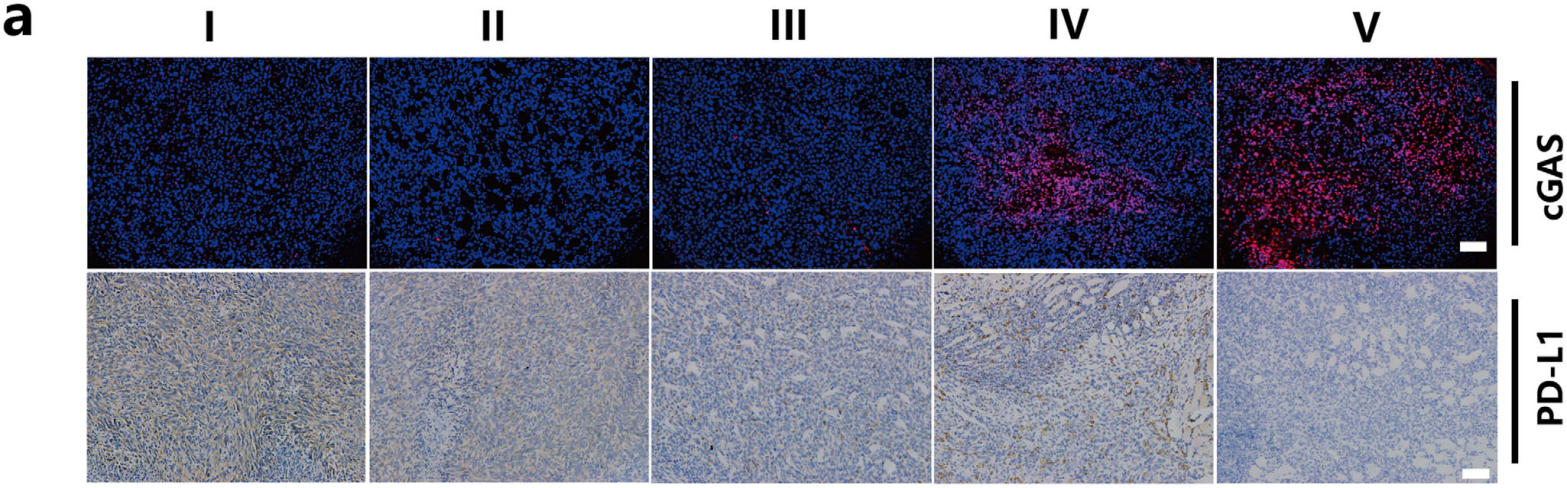


In Figure S22, the HE images of the liver in the PBS group and the heart in the PBS and ZH group were incorrect. Additionally, the spelling of “CZH” in the white inset of Figure S22 was corrected to “GCH.” The corrected figure is shown below.


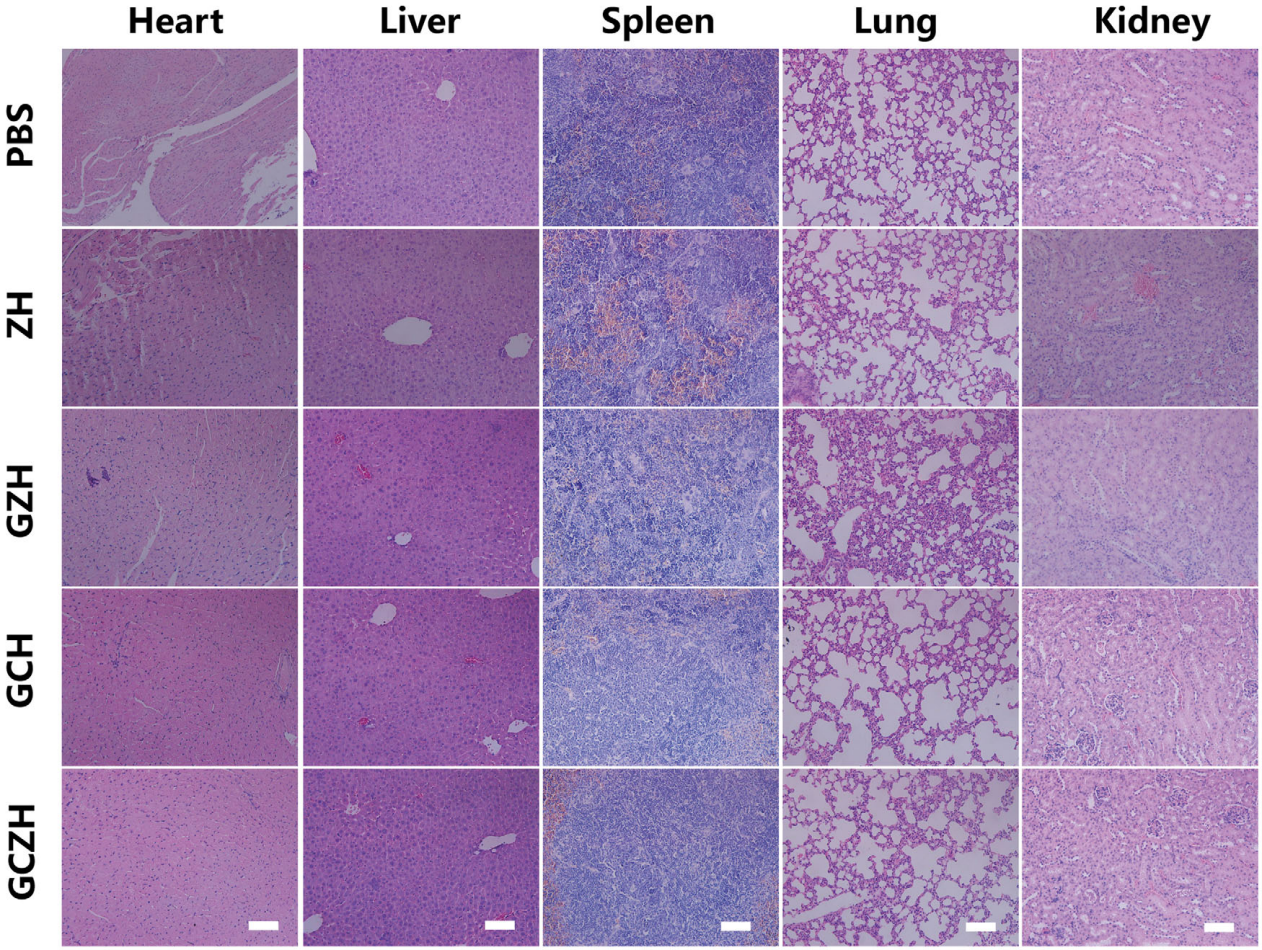


The corrections do not affect the overall findings and conclusions of this paper.

We apologize for these errors.

