# Supplementary material for: Advanced Cancer Immunotherapy via SMARCAL1 Blockade Using a Glucose‐Responsive CRISPR Nanovaccine
Source: Adv Sci (Weinh). 2025 Jul 12;12(37):e02929. doi: 10.1002/advs.202502929 (PMC12499461; doi:10.1002/advs.202502929)
Supplement: Supplementary file 1 — Supporting Information [file ADVS-12-e02929-s001.docx]

Supporting Information

**Advanced Cancer Immunotherapy via SMARCAL1 Blockade Using a Glucose‐Responsive CRISPR Nanovaccine**

*Yuwei Li ^†^, Yuanyi Zhang ^§^, Chenchen Li ^†^, Guoping Chen ^†^, Pir Muhammad ^†^, Yonghong Yao ^†^, Lifang Gao ^†^, Zhigang Liu ^‡^, Yanli Wang* ^†^*

Y. Li., C.Li., G.Chen., P. Muhammad ., Y.Yao, L.Gao., and Prof. Y.Wang

[†] Engineering Research Center of Tropical Medicine Innovation and Transformation of Ministry of Education, International Joint Research Center of Human-machine Intelligent Collaborative for Tumor Precision Diagnosis and Treatment of Hainan Province, Hainan Academy of Medical Sciences, Hainan Medical University, Hainan 571199, China.

E-mail: wangyanli@muhn.edu.cn
[§] Y. Zhang.

Department of Immunology & Key Laboratory of Tropical Translational Medicine of Ministry of Education, School of Basic Medicine and Life Sciences, Hainan Medical University, Hainan 571199, China.

[‡] Z. Liu

State Key Laboratory of Respiratory Disease for Allergy, School of Medicine, Shenzhen University, Shenzhen 518060, China.


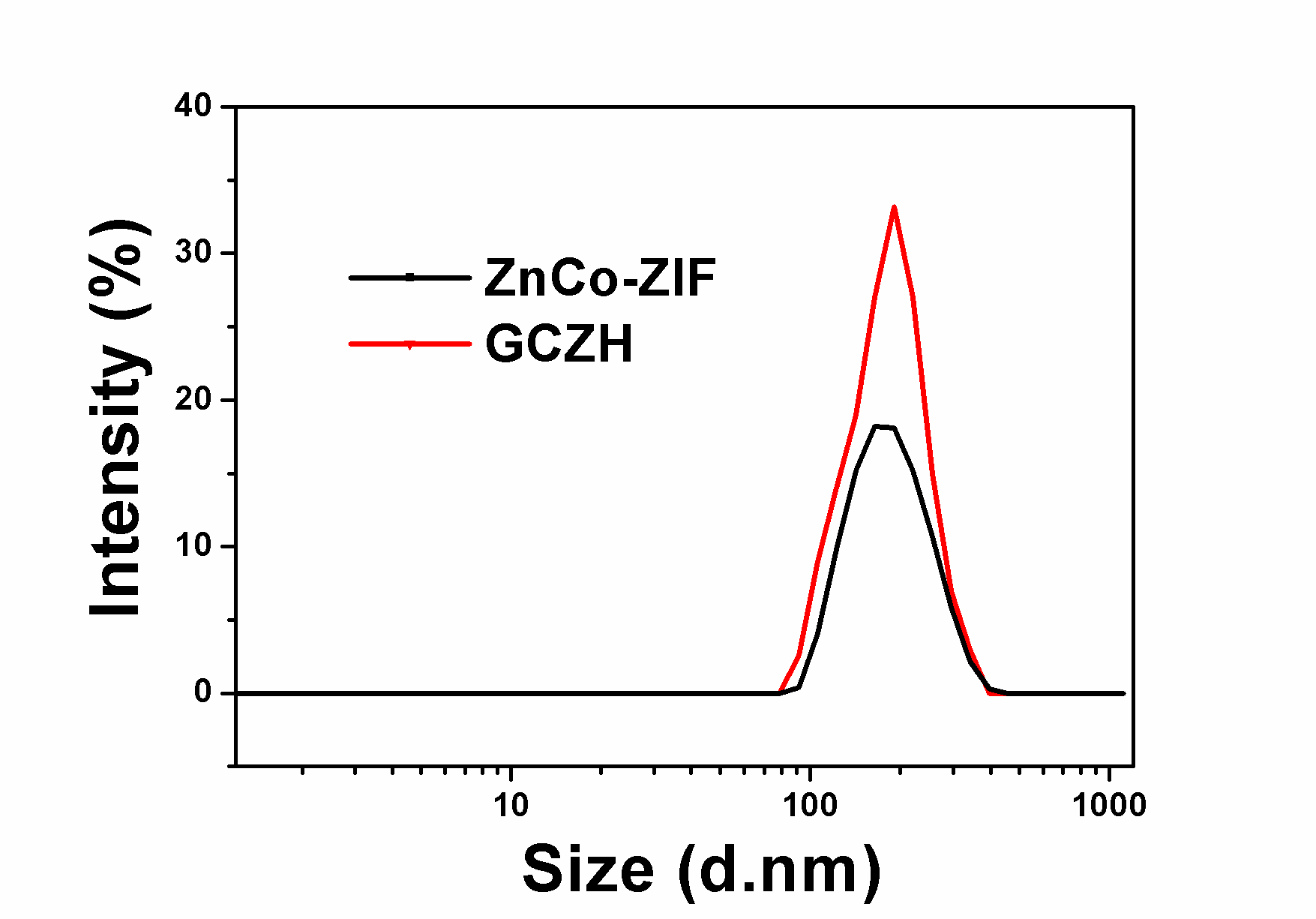


**Figure S1.** The size distribution of the ZnCo-ZIF and GCZH.


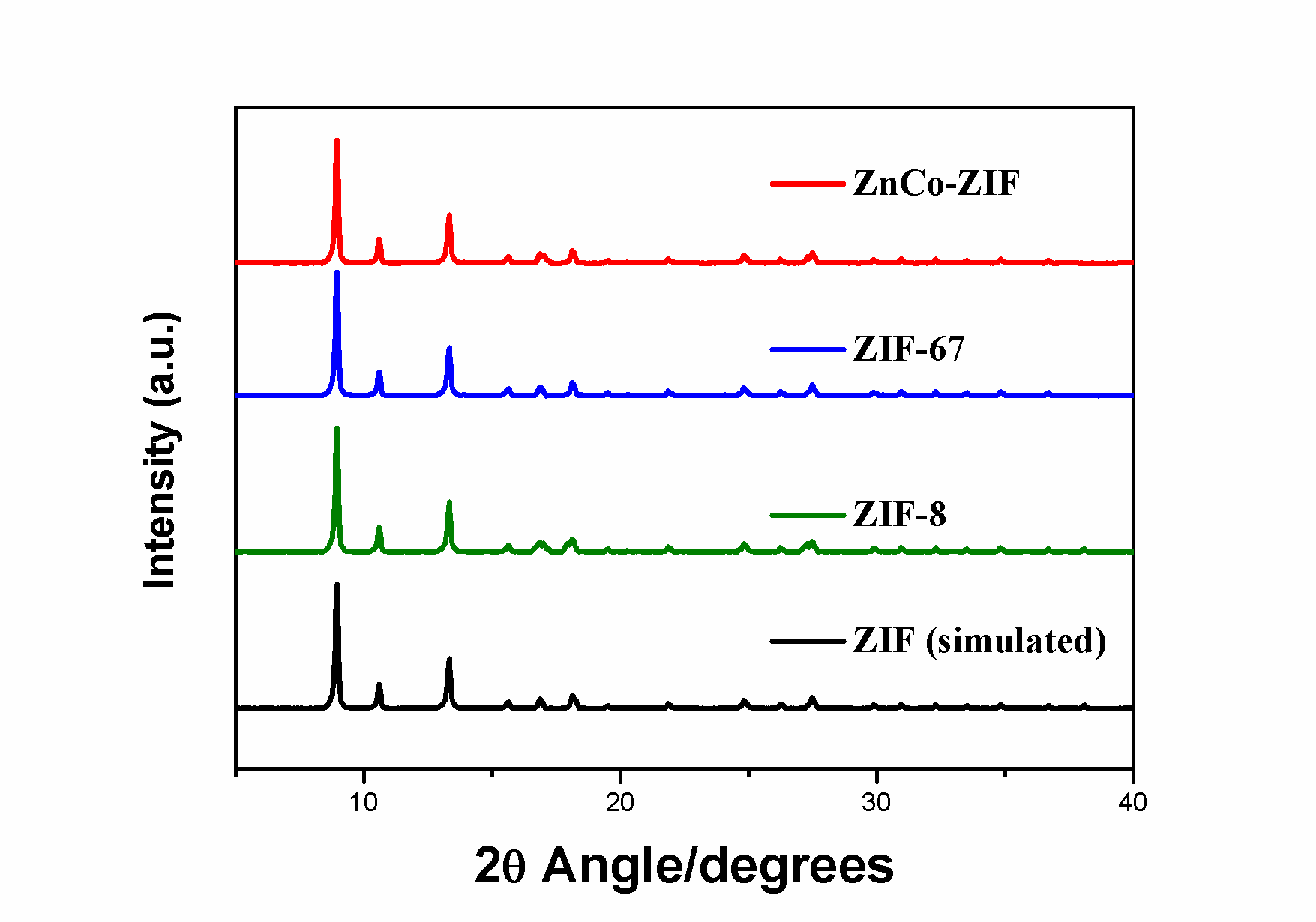


**Figure S2**. XRD patterns of the ZIF-8, ZIF-67 and ZnCo-ZIF nanoparticles.


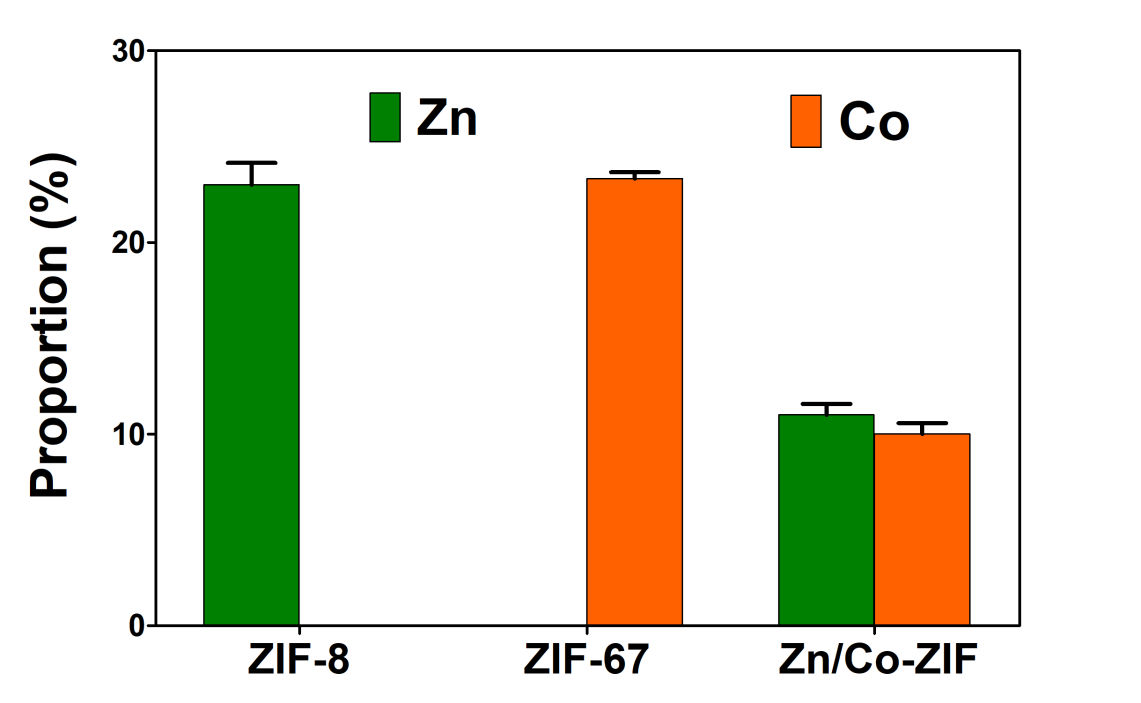


**Figure S3.** The zinc and cobalt content of the ZIF-8, ZIF-67 and ZnCo-ZIF nanoparticles. Data were presented as the means ± SD (n = 3).


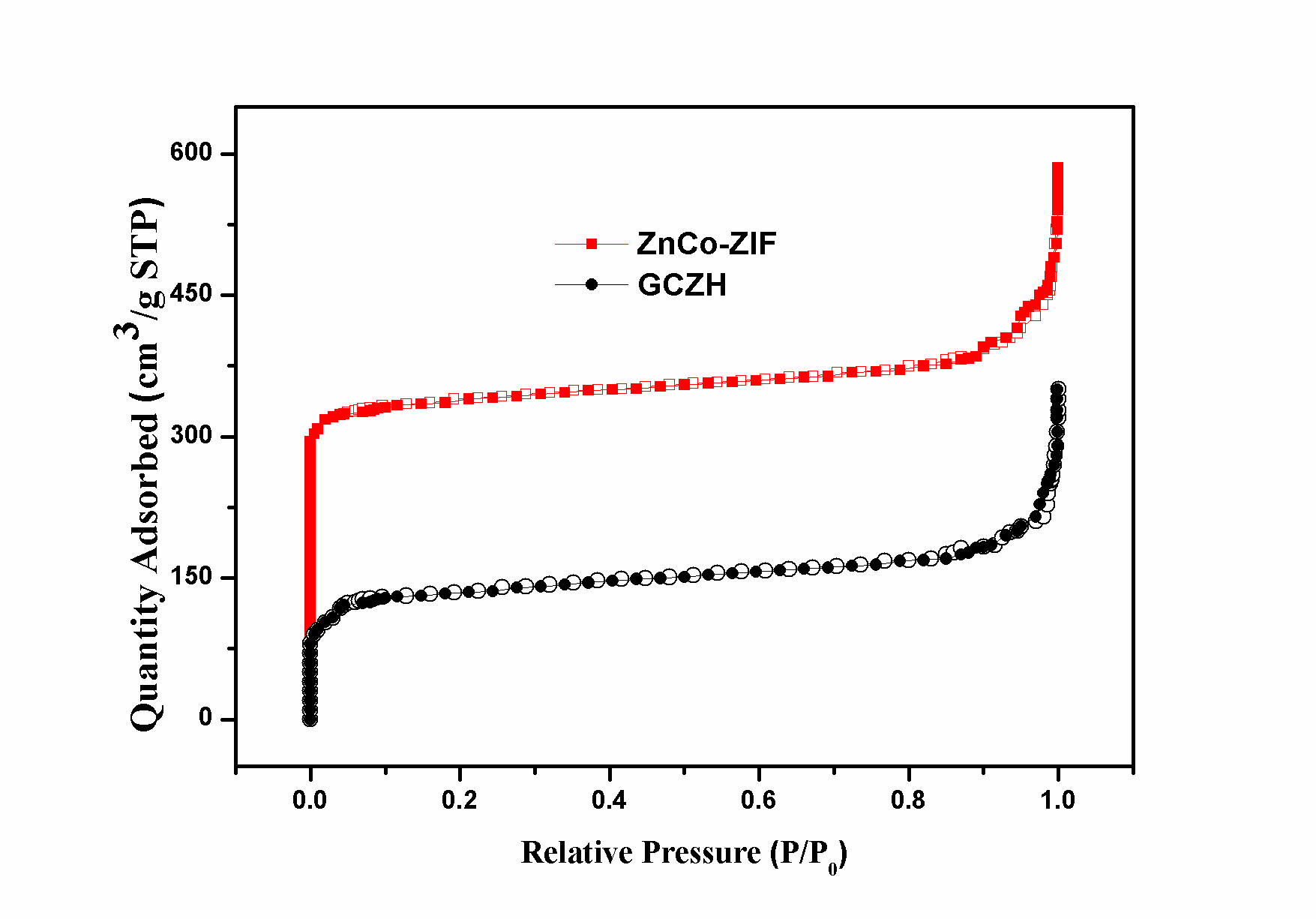


**Figure S4.** N_2_ adsorption–desorption isotherms of the ZnCo-ZIF and GCZH nanoparticles.


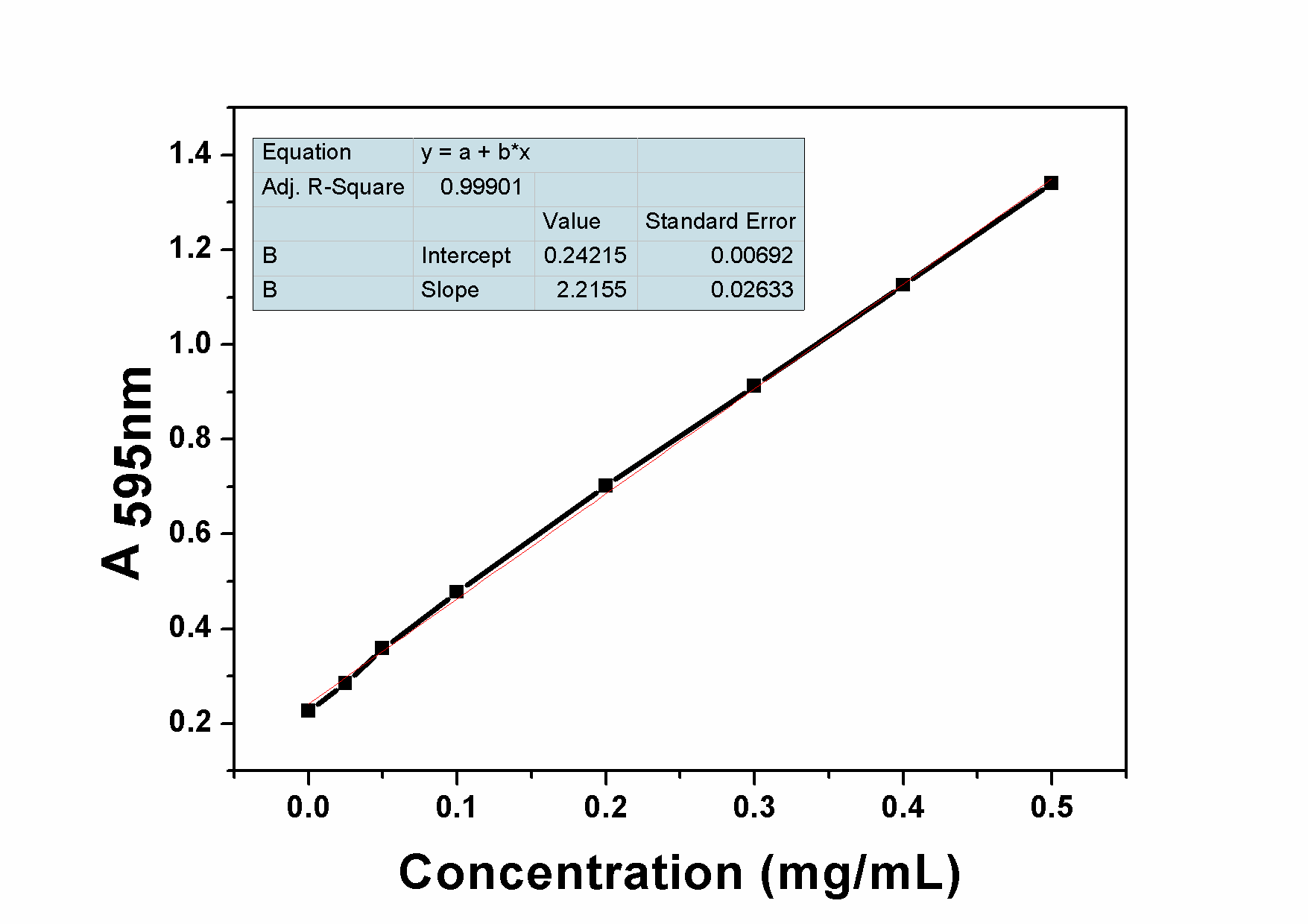


**Figure S5.** Linear relationships between the UV-vis absorbance intensity at 595 nm and the concentration of GCZH.


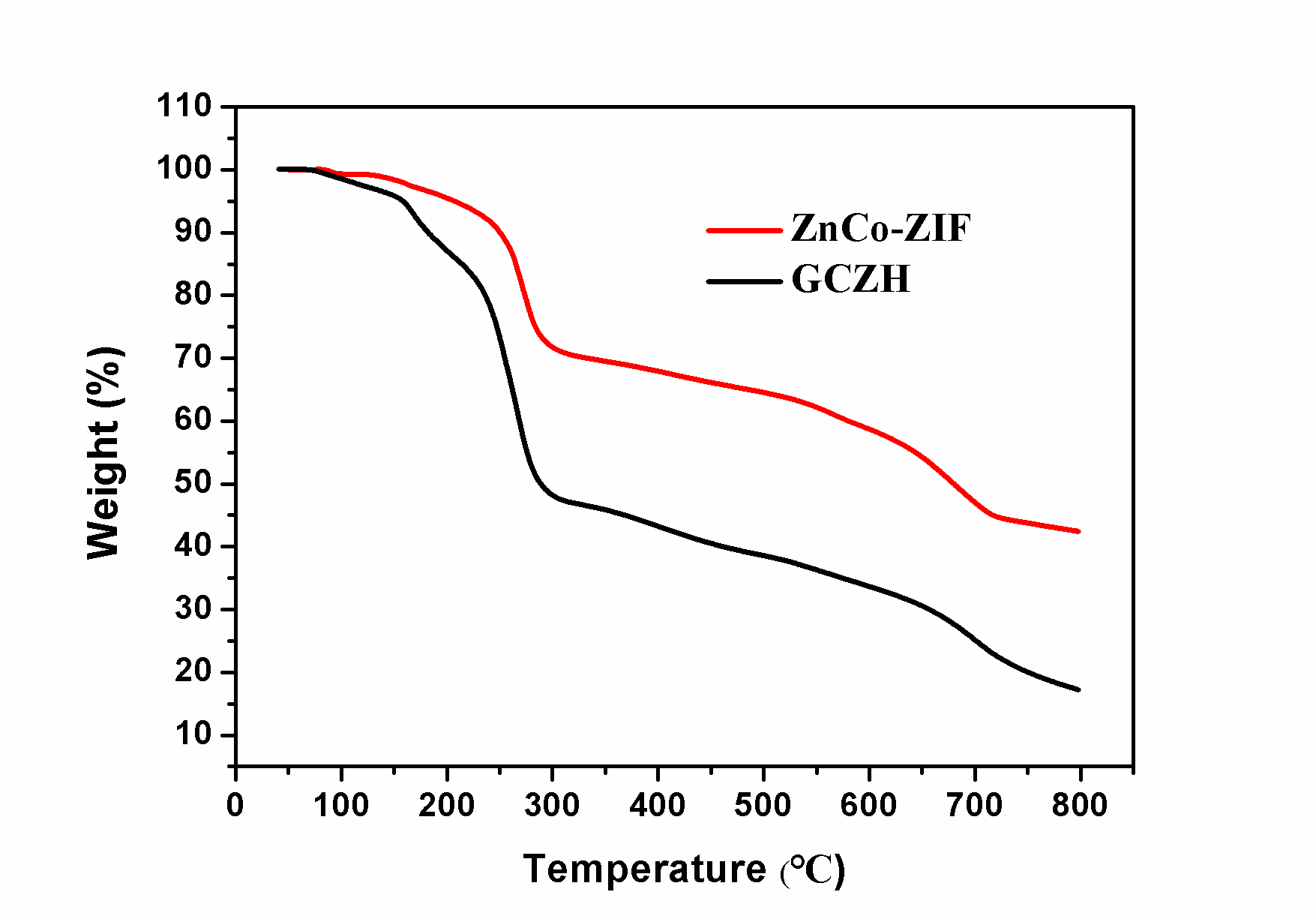


**Figure S6.** Thermo gravimetric analysis of the ZnCo-ZIF and GCZH nanoparticles in air.


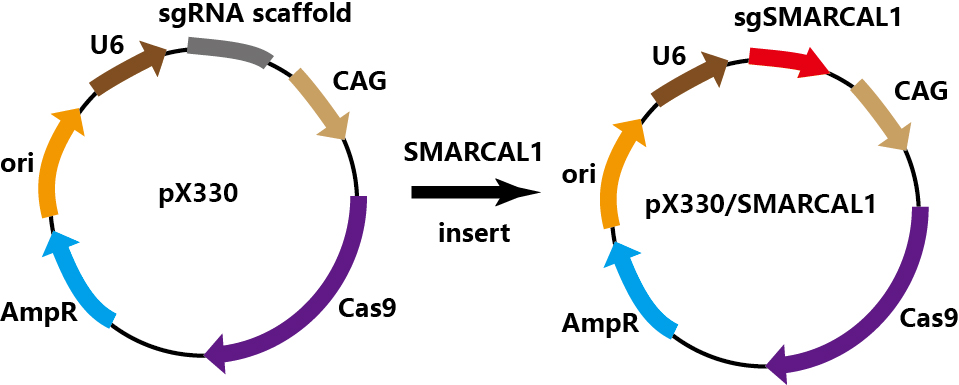


**Figure S7.** Validation of the constructed plasmid for SMARCAL1 knockout.


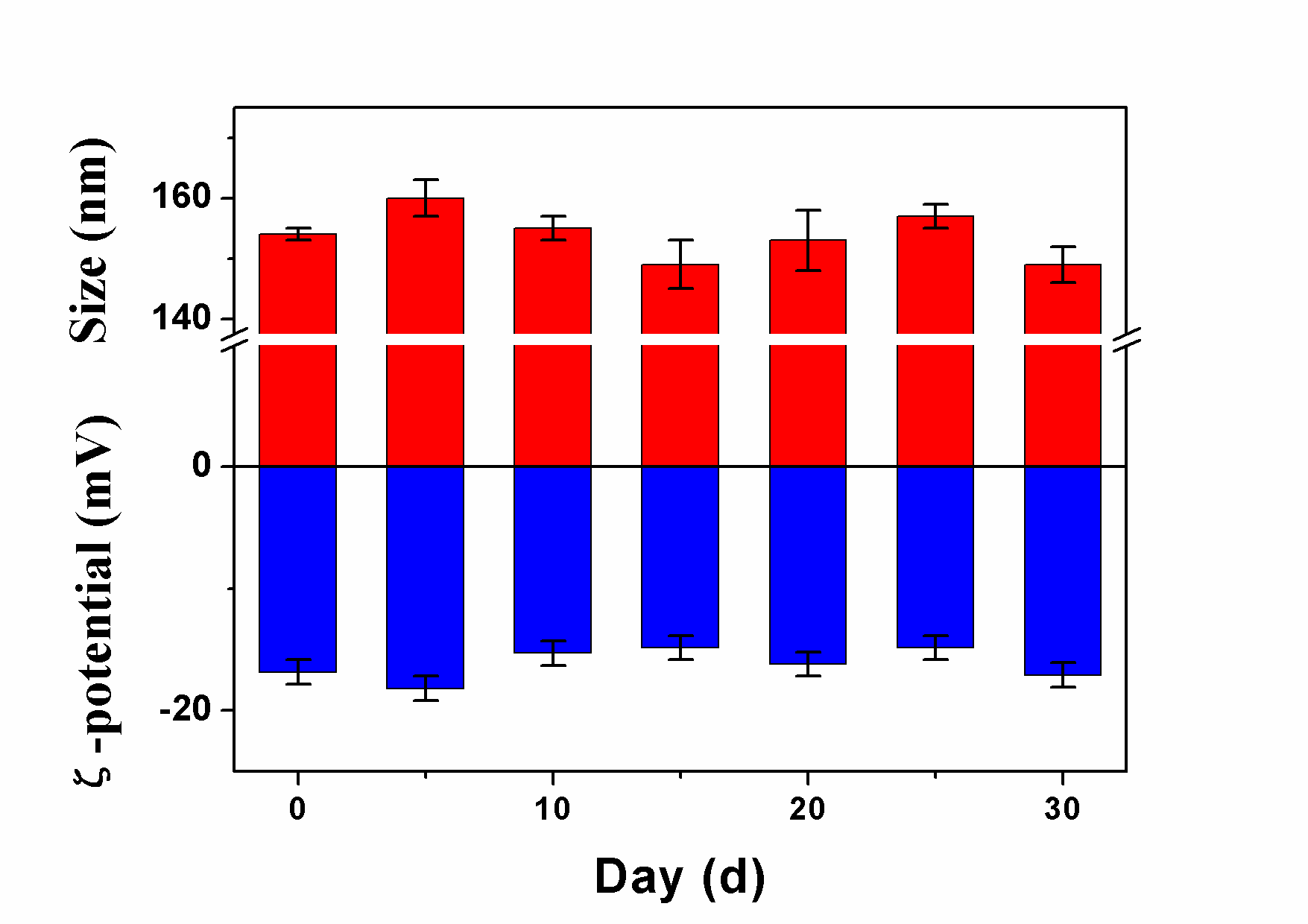


**Figure S8.** Colloidal stability of the GCZH NPs. Average size and zeta potential of the GCZH NPs were measured over 30 days, when stored at 4 ºC. Data are presented as the means ± SD (n = 3).


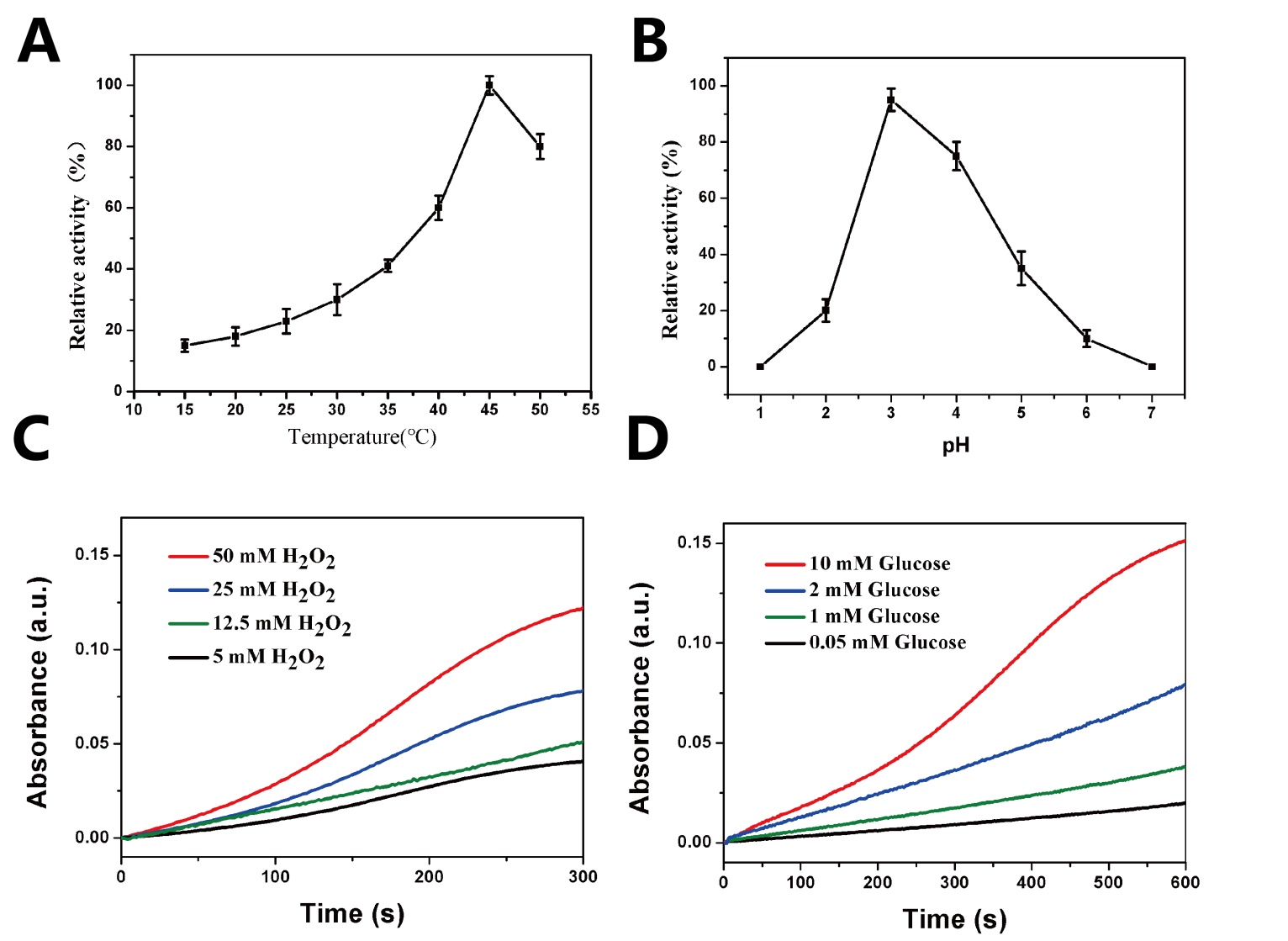


**Figure S9.** Peroxidase-like catalytic activity of GCZH is dependent on (A) temperature, (B) pH, (C) H_2_O_2_ and (D) glucose concentration. (A**–**B) Experiments were performed using 96 μg mL^-1^ GCZH in a reaction volume of 500 μL in 25 mM NaAc buffer (pH=3), and 20 mM H_2_O_2_, with 1.2 mM TMB as substrate under different temperature (A) or pH (B) (mean ± SD, n=3). (C**–**D) Experiments were performed using 96 μg mL^-1^ GCZH in a reaction volume of 500 μL in 25 mM NaAc buffer (pH=3.0, 37°C), different concentration of H_2_O_2_ (C) or glucose (D) with 1.2 mM TMB as substrate..


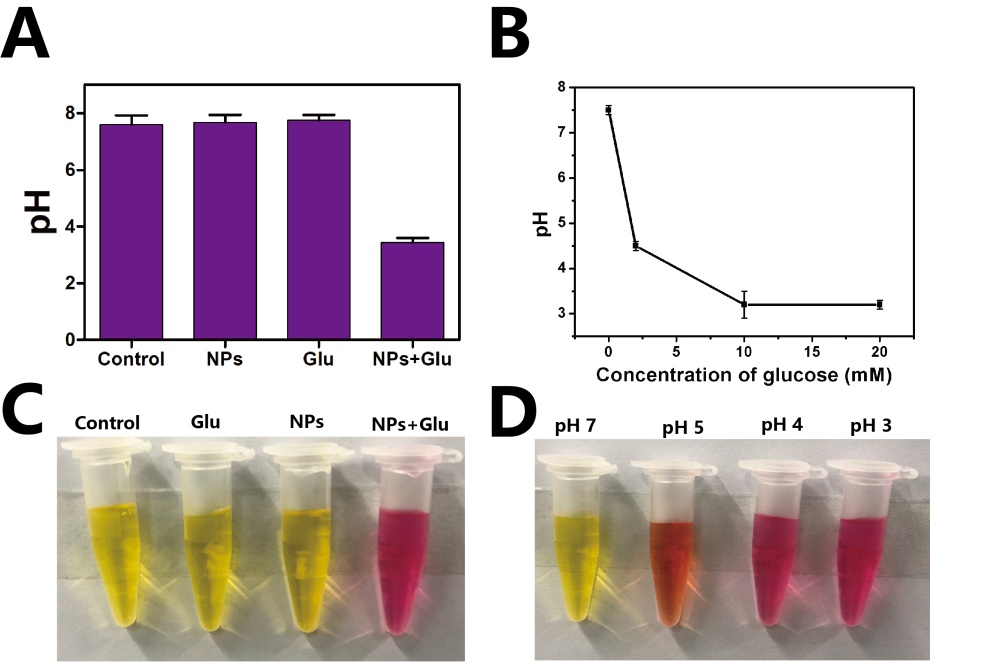


**Figure S10.** (A) The pH value of different reaction system in NaAc buffer (glucose=10 mM, mean ± SD, n=3). (B) Variation of pH value arising from the reaction between GCZH and different concentrations of glucose (mean ± SD, n=3). (C) Corresponding photographs of different reaction system in (A) upon the addition of methyl red. (D) Standard photographs of phosphate buffers at different pH values upon the addition of methyl red. The reactions in (A**–**C) were recorded after 1 h incubation.


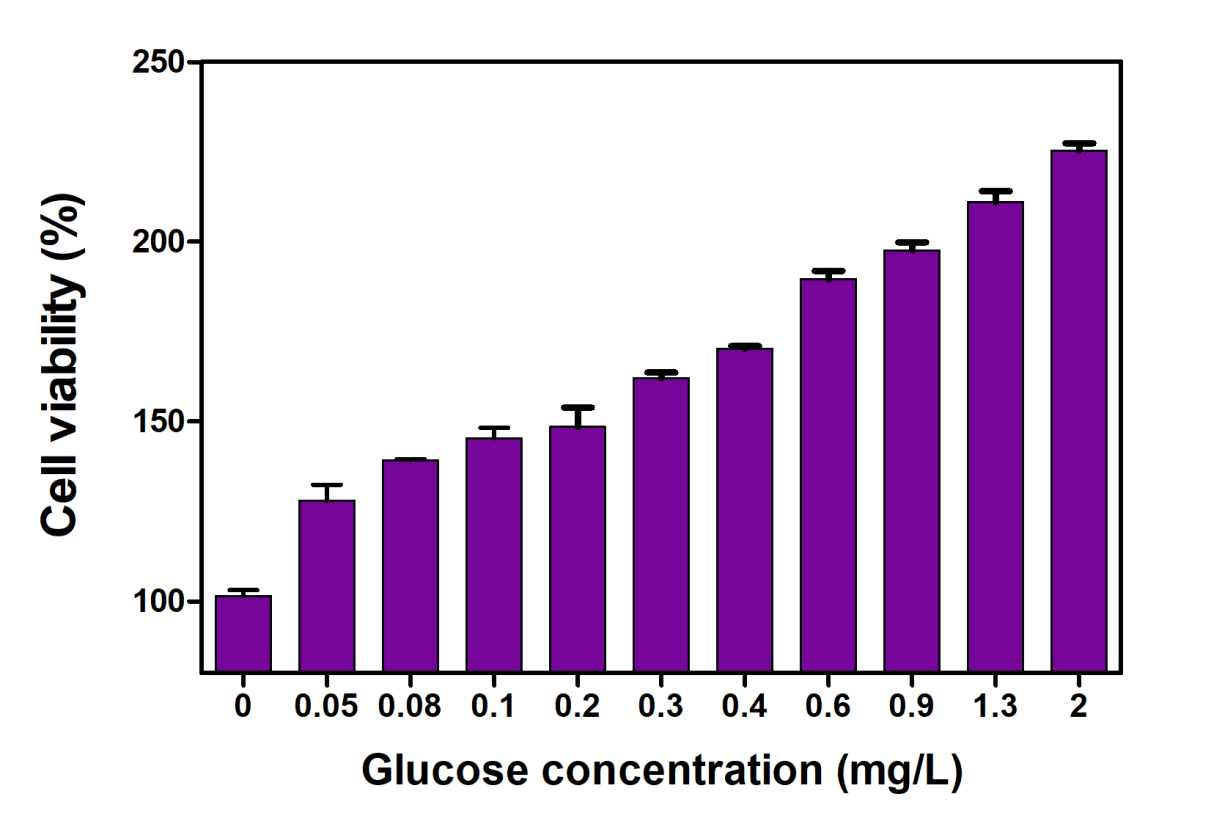


**Figure S11.** The cell viability of 4T1 cells after treatment with various concentrations glucose for 24 h. Data are presented as the means ± SD (n = 3).


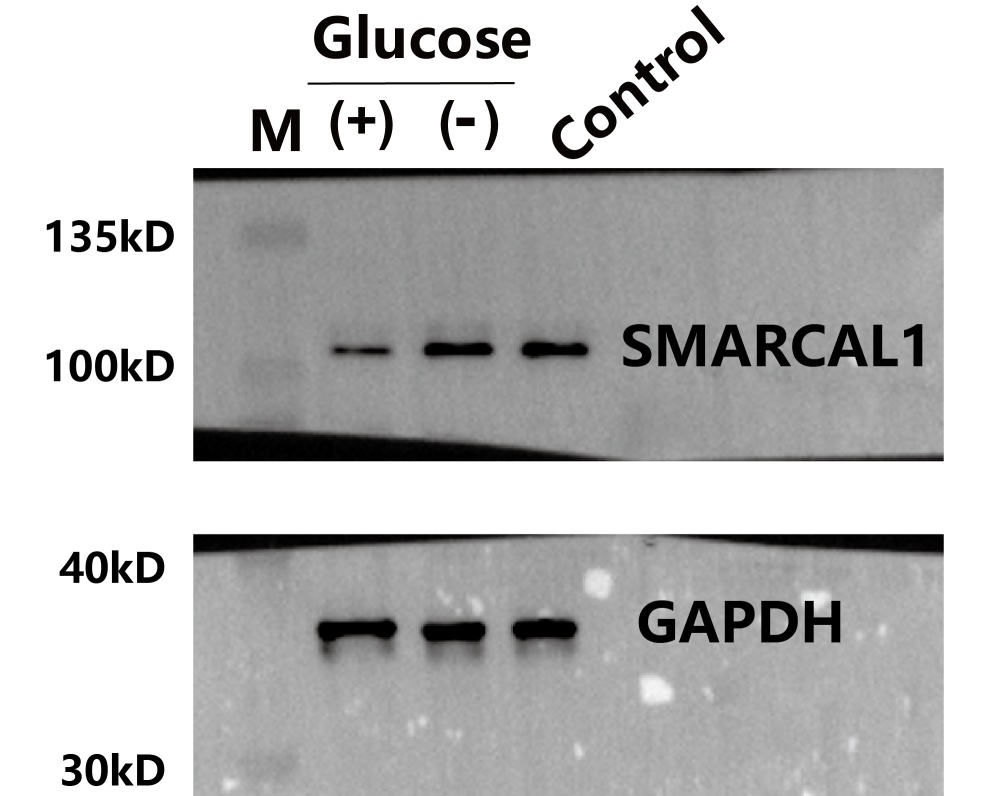


**Figure S12**. Western blot analysis on expression of SMARCAL1 in 4T1 after different treatments. The amount of protein loading was evaluated by GAPDH.

**
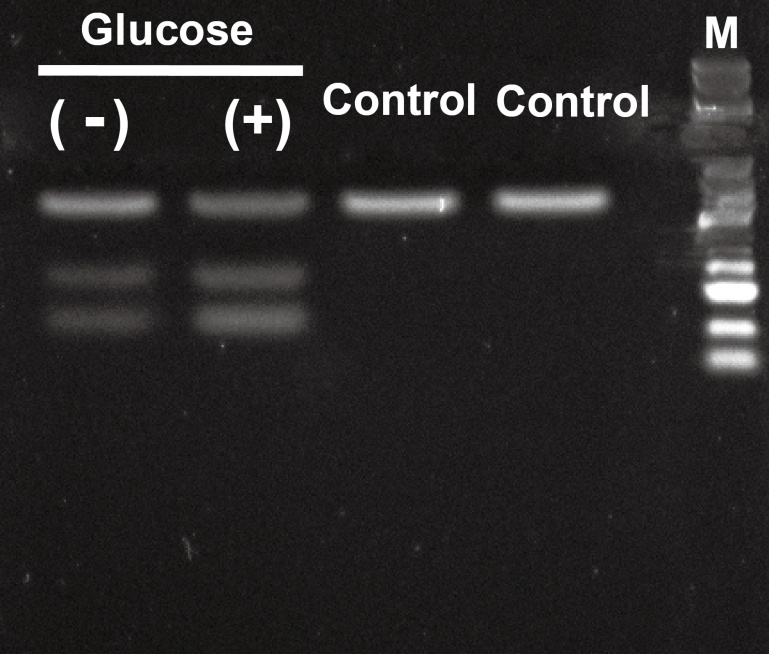
**

**Figure S13**. T7EI assay of SMARCAL1 gene disruption in different treatment groups.


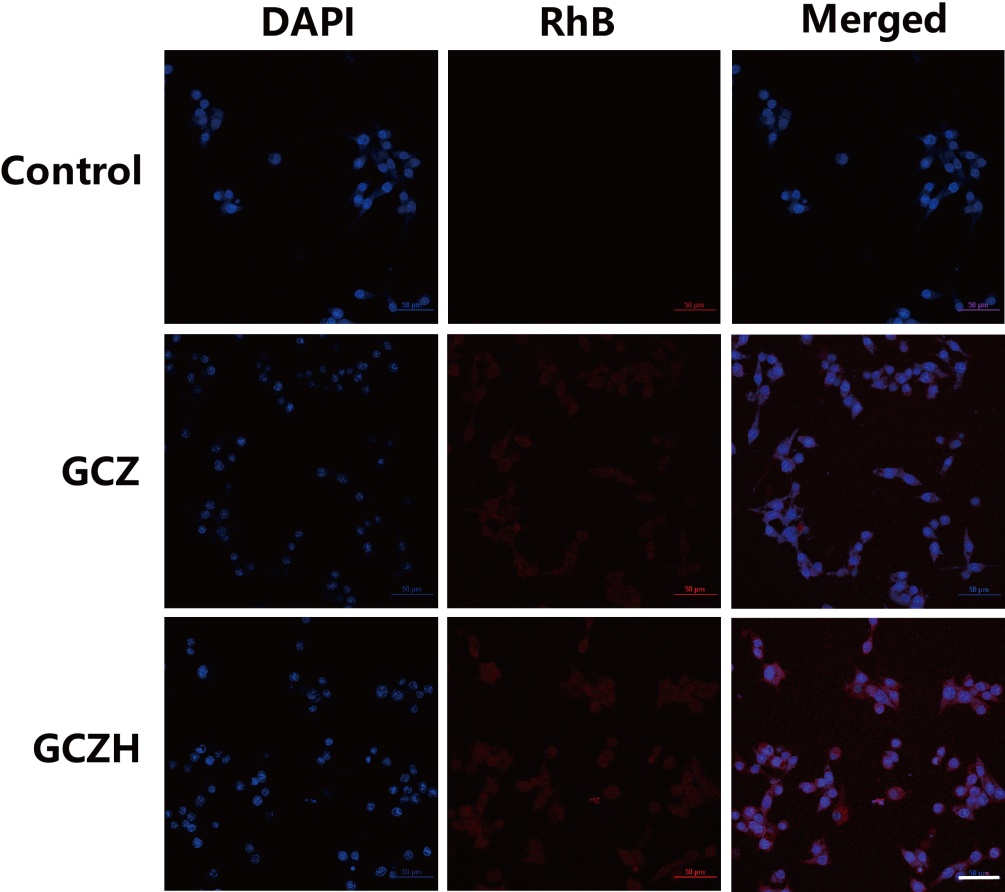


**Figure S14.** Fluorescence micrographs analysis of cellular uptakes against RhB-labeled nanoparticles. Scale bars are 50 μm.


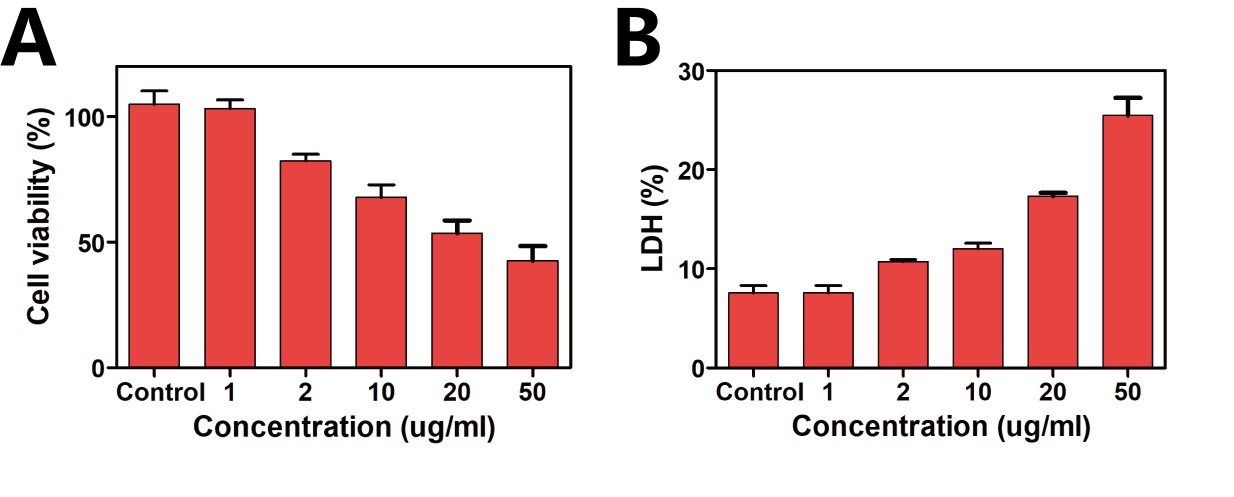


**Figure S15.** Cell viability (A), and specific lysis (B) of 4T1 cells treated by different concentration GCZH under 1 mM glucose condition. Data are presented as the means ± SD (n = 3).


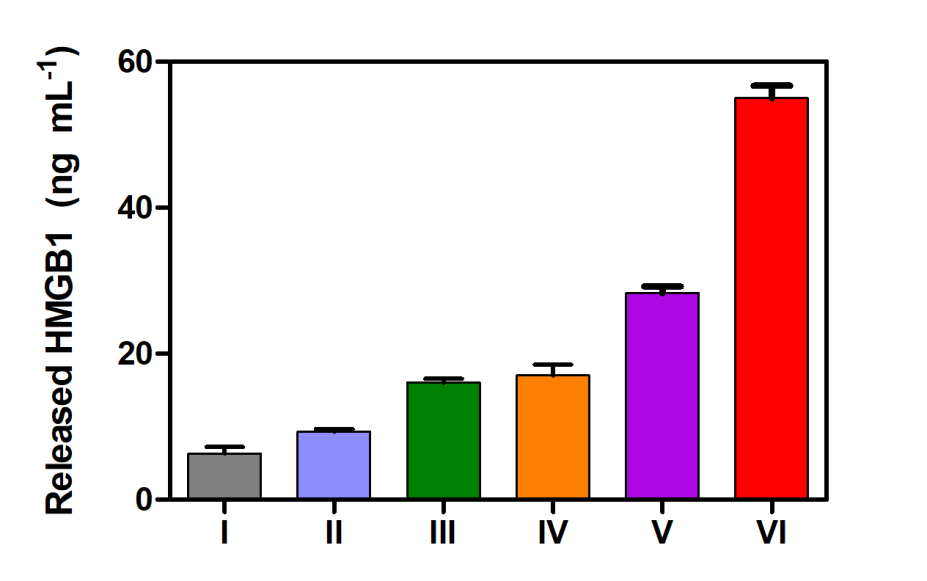


**Figure S16.** The concentration levels of HMGB1 in the supernatant of the 4T1 cells after treatment with different groups. Data are presented as the means ± SD (n = 3).


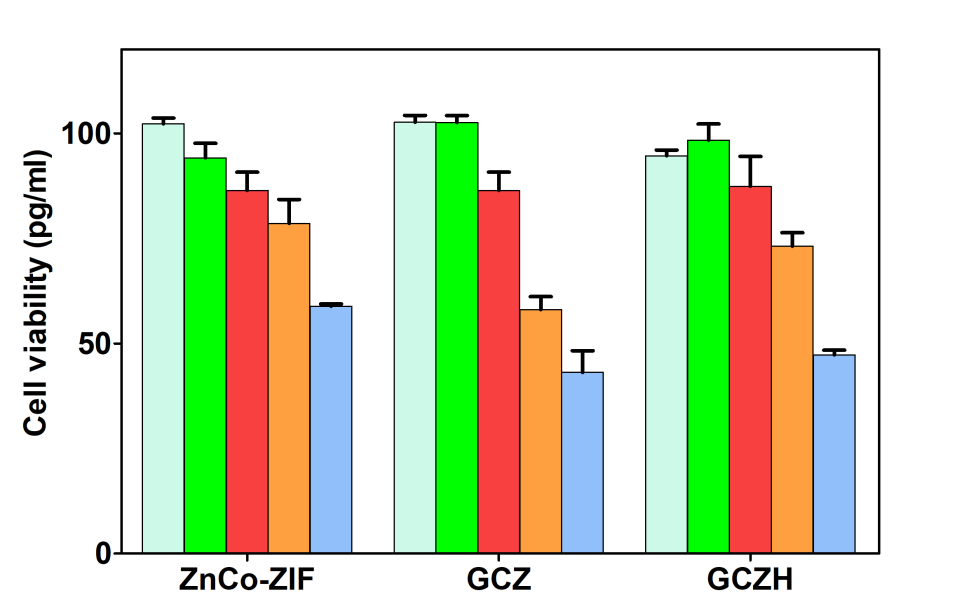


**Figure S17.** Cytotoxicity of nanoparticles on NIH 3T3 cells that were incubated with different concentrations (0, 20, 50, 100, and 200 μg/ml, from left to right) of nanoparticles for 24 h. Data are presented as mean ± SD (n = 3).


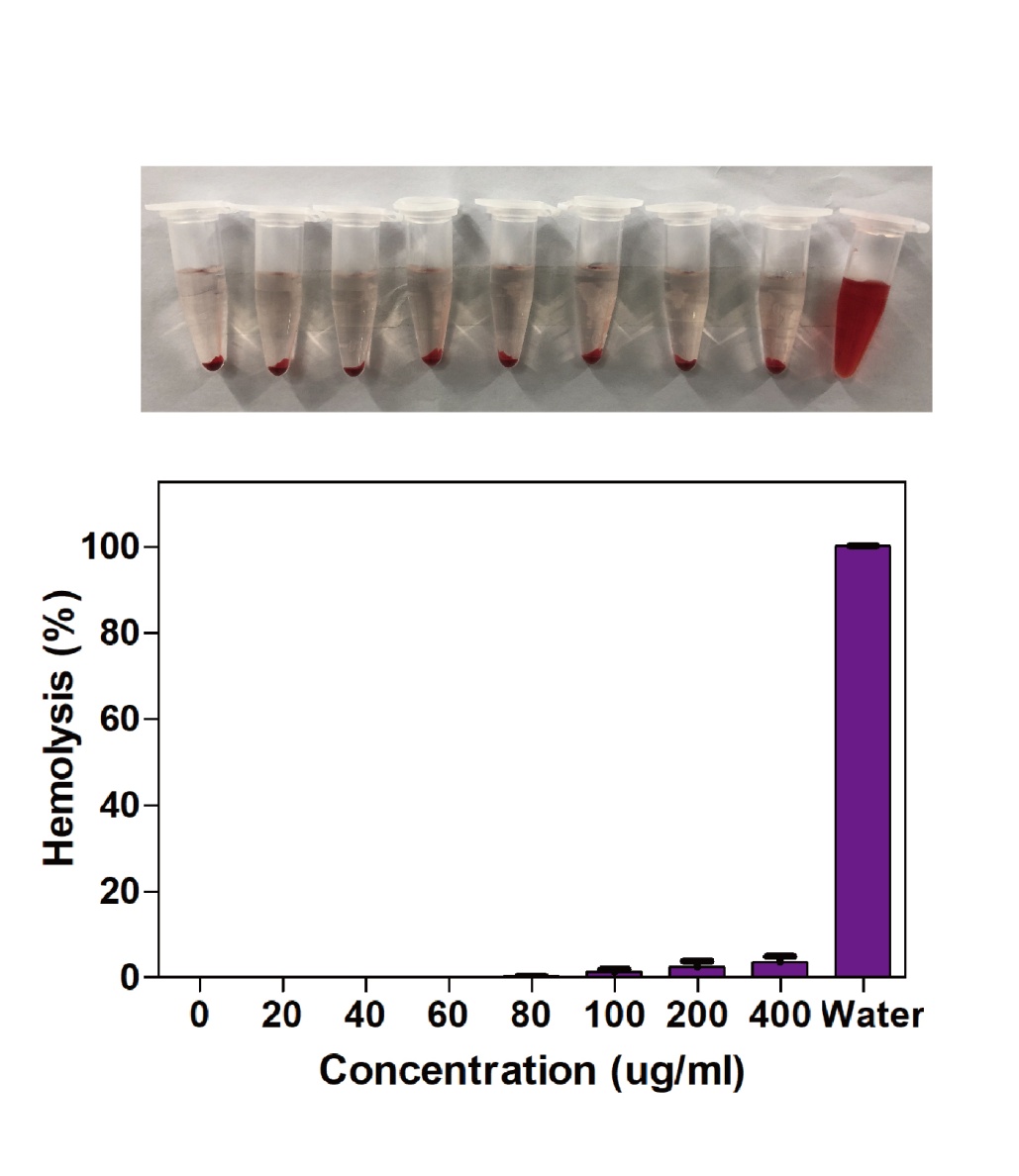


**Figure S18.** (A) Digital photograph of hemolysis test and (B) the hemolysis rate (HR%) of GCZH nanoparticles. Data are presented as the means ± SD (n = 3).


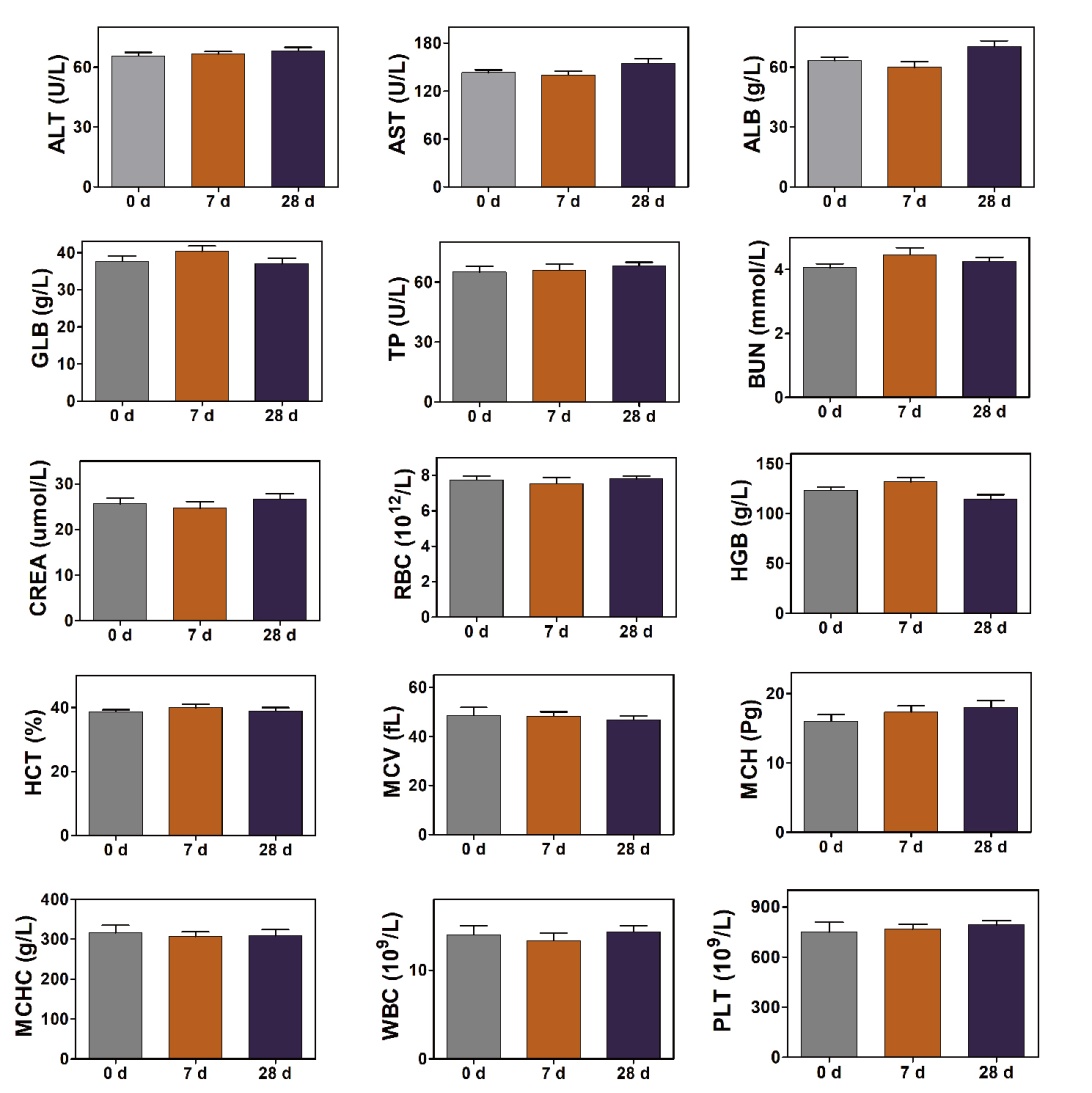


**Figure S19.** *In vivo* toxicity. Blood biochemical and hematological analysis of the healthy mice intravenously injected with nanoparticles at 7 and 28 days’post-injection. ALT, alanine transferase; AST, aspartate transferase; ALB, alburnin; GLB, globulin; TP, total protein; BUN, blood urea nitrogen; CREA, creatinine; RBC, red blood cells; HGB, hemoglobin; HCT, hematocrit; MCV, mean capsular volume; MCH, mean capsular hemoglobin; MCHC, mean capsular hemoglobin concentration; WBC, white blood cells, PLT, platelets. Data are presented as the means ± SD (n = 5).


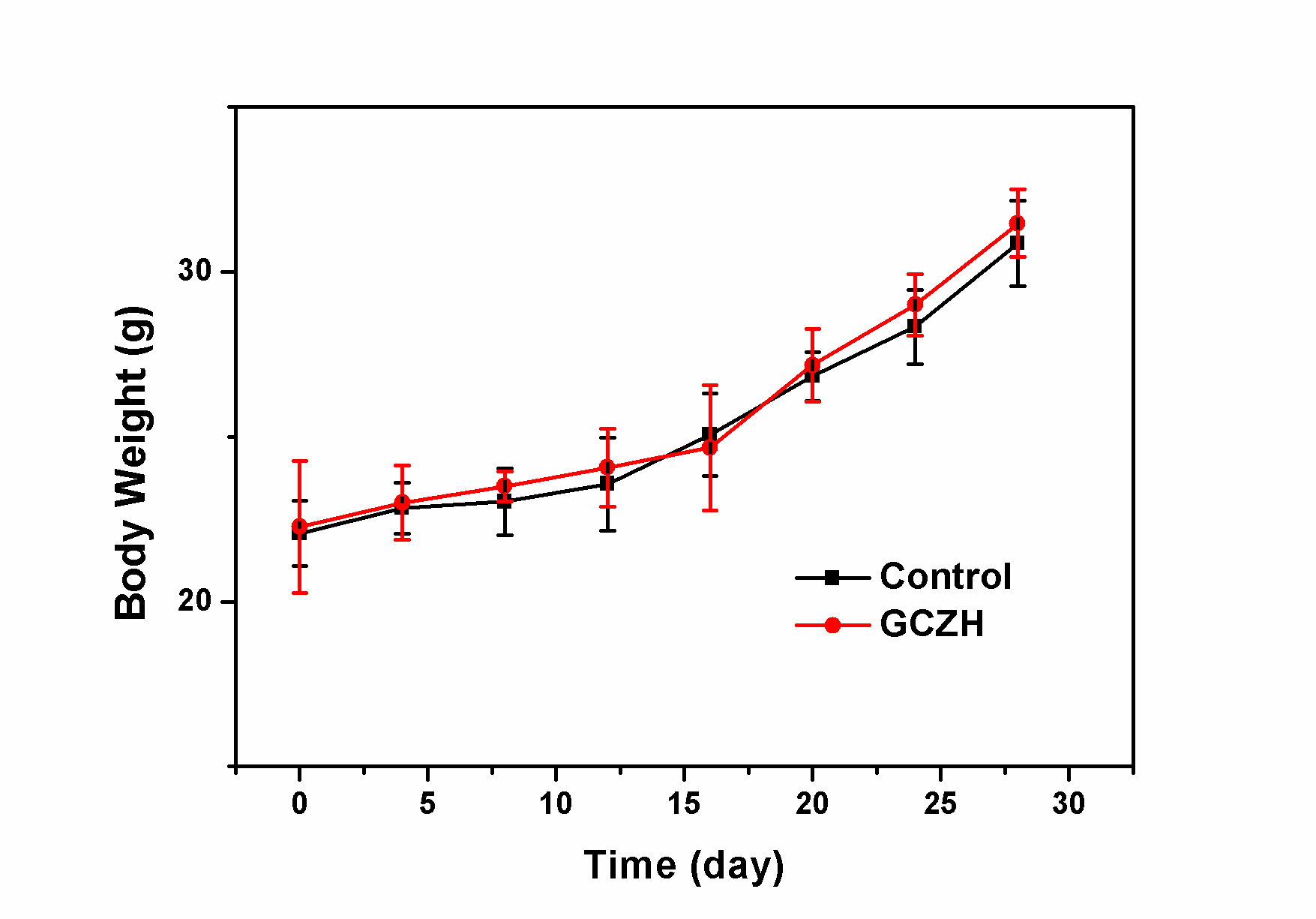


**Figure S20.** Body weights of mice treated by GCZH were measured every 4 days to evaluate the *in vivo* biosafety. Data are presented as the means ± SD (n = 3).


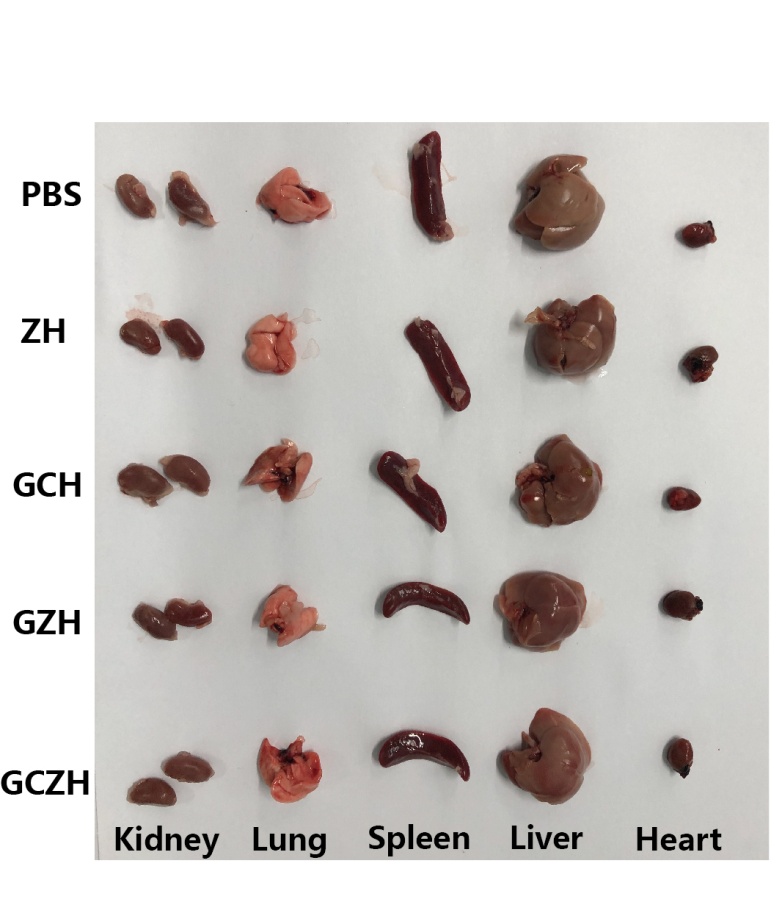


**Figure S21.** The major organs of at 28 days post the injection. No abnormalities are observed in major organs comparing with the one of control group.


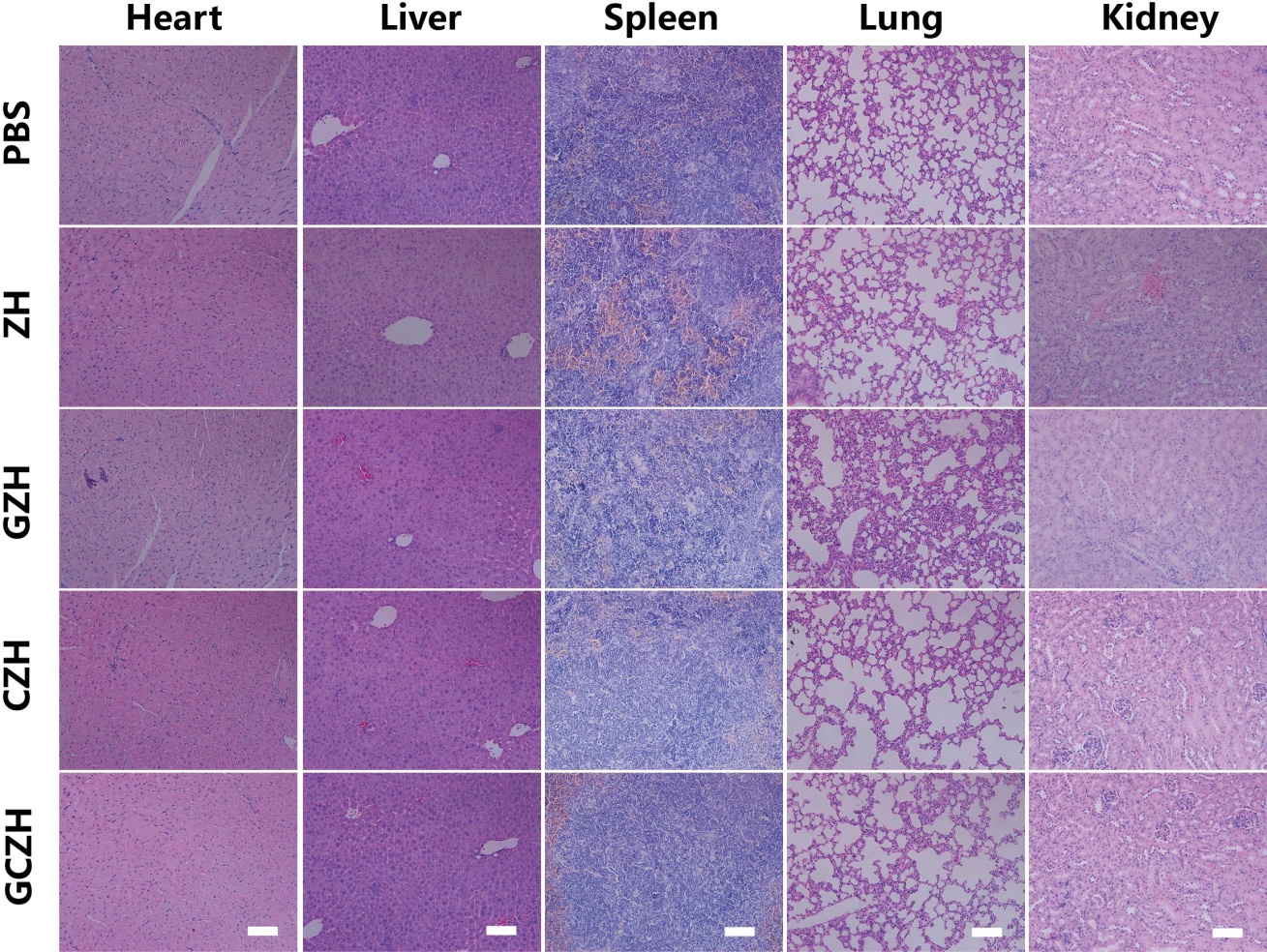


**Figure S22.** H&E stained images of major organs at 28 days post the injection. No abnormalities are observed in major organs comparing with the one of control group. (Scale bar= 100 μm)


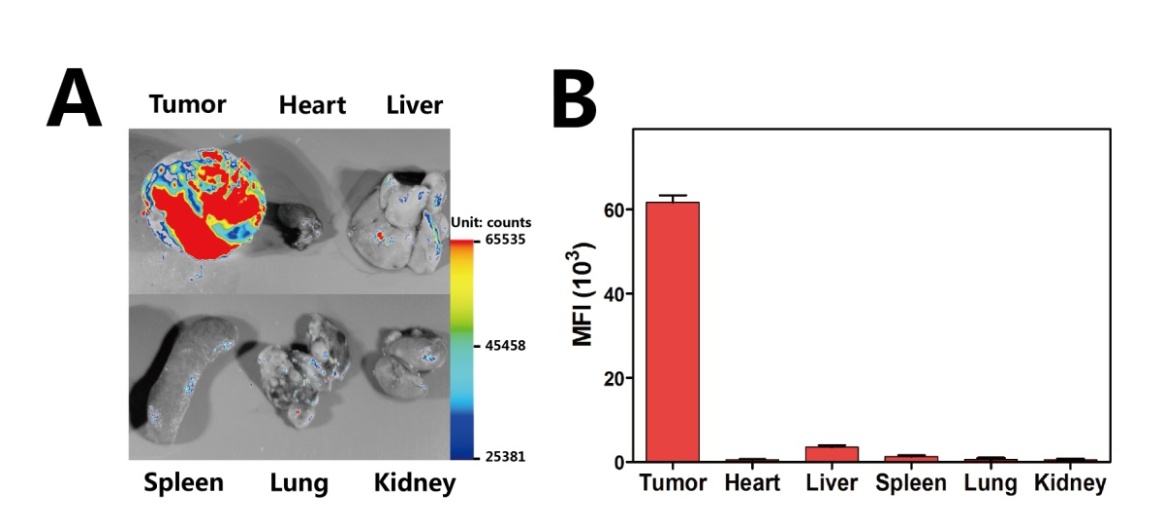


**Figure S23.** (A) *Ex vivo* fluorescence images of the tumors and major organs harvested at 12 h post-intravenous injection of Cy3-labeled GCZH. (B) Mean fluorescence intensity (MFI) in (A) (mean ± SD, n = 3).


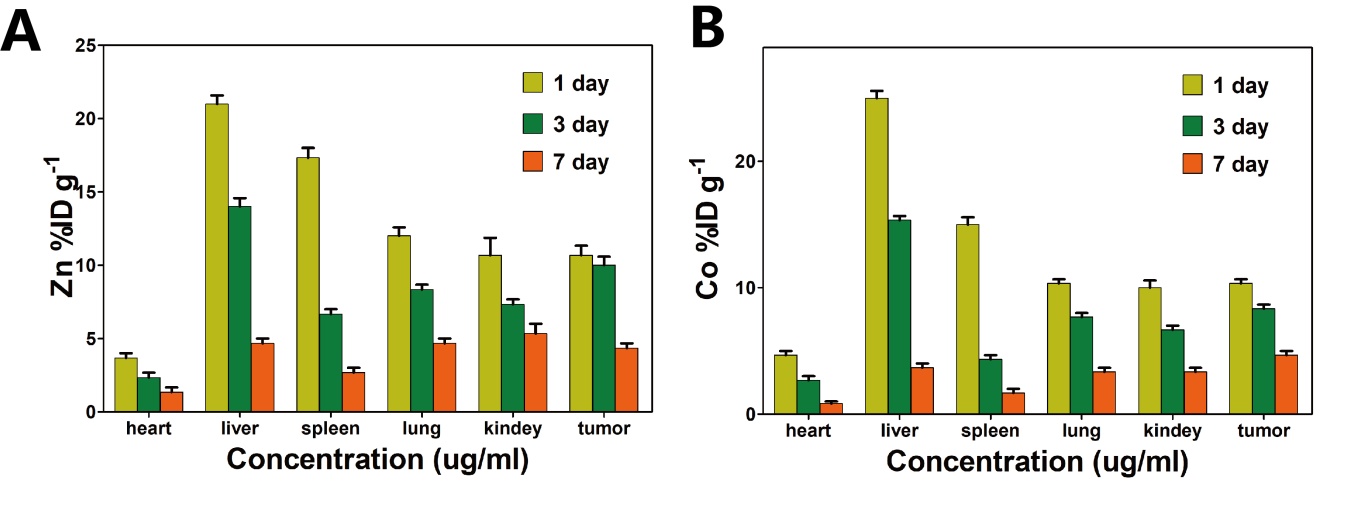


**Figure S24.** Biodistribution of Zinc (Zn) and cobalt (Co) ions in main organs at different time points. Data are shown as the means ± SD (n = 3).
